# Whole-Exome Sequencing Identifies Genetic Variants for Severe Adolescent Idiopathic Scoliosis in a Taiwanese Population

**DOI:** 10.3390/jpm13010032

**Published:** 2022-12-23

**Authors:** Min-Rou Lin, Po-Hsin Chou, Kuei-Jung Huang, Jafit Ting, Chia-Ying Liu, Wan-Hsuan Chou, Gan-Hong Lin, Jan-Gowth Chang, Shiro Ikegawa, Shih-Tien Wang, Wei-Chiao Chang

**Affiliations:** 1 Department of Clinical Pharmacy, School of Pharmacy, Taipei Medical University, Taipei 110, Taiwan; 2 Department of Orthopedics and Traumatology, Taipei Veterans General Hospital, Taipei 112, Taiwan; 3 School of Medicine, National Yang Ming Chiao Tung University, Taipei 112, Taiwan; 4 Master Program in Clinical Genomics and Proteomics, School of Pharmacy, Taipei Medical University, Taipei 110, Taiwan; 5 Center for Precision Medicine, China Medical University Hospital, Taichung 404, Taiwan; 6 School of Medicine, China Medical University, Taichung 404, Taiwan; 7 Laboratory for Bone and Joint Diseases, RIKEN Center for Integrative Medical Science (IMS, RIKEN), Tokyo 108-8639, Japan; 8 Kinmen Hospital, Ministry of Health and Welfare, Kinmen 891, Taiwan; 9 Integrative Research Center in Critical Care, Wan Fang Hospital, Taipei Medical University, Taipei 116, Taiwan

**Keywords:** adolescent idiopathic scoliosis, whole-exome sequencing, TTN, CLCN1, SOX8

## Abstract

Adolescent idiopathic scoliosis (AIS) is a three-dimensional spinal curvature deformity that appears in the adolescent period. In this study, we performed whole-exome sequencing on 11 unrelated Taiwanese patients with a Cobb’s angle greater than 40 degrees. Our results identified more than 200 potential pathogenic rare variants, however, most of which were carried only by one individual. By in silico pathogenicity annotation studies, we found that *TTN*, *CLCN1*, and *SOX8* were the most important genes, as multiple pathogenic variants were within these genes. Furthermore, biological functional annotation indicated critical roles of these AIS candidate genes in the skeletal muscle. Importantly, a pathogenic variant on *SOX8* was shared by over 35% of the patients. These results highlighted *TTN*, *CLCN1*, and *SOX8* as the most likely susceptibility genes for severe AIS.

## 1. Introduction

Adolescent idiopathic scoliosis (AIS) is a three-dimensional deformity spinal curvature that appears in adolescence with a prevalence of 0.47–5.2% [1]. Scoliosis is characterized by deviation of the spine with a Cobb angle greater than 10 degrees. A spine with a Cobb angle greater than 40 degrees usually cause serious complications including lungs and heart damages. While the progression of scoliosis tends to ease up when patients stop growing, large-curve scoliosis frequently continues to worsen throughout adulthood [2]. Symptoms of AIS include asymmetry in the shoulder, waist, or hip, or functional leg length discrepancy. In addition, the worldwide prevalence as well as curve severity of AIS, is greater in females than males [1]. However, the etiology and the underlying mechanisms of sexual dimorphism are still unclear.

The diagnosis of AIS can be made by using a forward-bending test with or without a scoliometer. Anterior–posterior and lateral radiographs are applied to further confirm the angle of the curvature and to monitor disease progression. For the measurement of rotation, three-dimensional CT scans and magnetic resonance imaging were used to reconstruct the spine [3]. AIS symptoms can be mitigated in numerous ways. Surgical treatments are widely applied for patients with severe curves. For mild scoliosis, brace therapy is often combined with physiotherapy. However, the use of braces might subsequently affect development of the heart and lungs in the thoracic cavity [4]. Over-treating a child with a long-term brace prescription might cause social, financial, and psychological problems [5]. Alternative treatments such as chiropractic medicine and electrical stimulation have been suggested to improve patients’ quality of life; however, the benefits for prevention of scoliosis progression remain controversial [6].

Genetic factors such as single-nucleotide variant (SNV) and copy number variants (CNV) have been recognized as potential causes of AIS [7]. Genome-wide association studies (GWASs) have revealed *LBX1* [8], *GPR126* [9], and *BNC2* [10] as the susceptibility genes of AIS. In an exome sequencing study of 91 severe AIS patients, *FBN1* and *FBN2* were identified as candidate genes [11]. Haller et al, by analyzing 391 severe AIS cases and 843 controls [12], found that AIS is associated with extracellular matrix genes, *COL11A2*. Previous research has investigated the genetic susceptibility to low bone mass and osteopenia reported in AIS patients. A case–control study has revealed that genetic polymorphisms in *ADIPOQ* are associate with AIS osteopenia through the dysregulation of *ADR1-RANKL/OPG* and *ADR1-IL6* pathways [13]. In addition, *BSML* polymorphism was reported to associate with the lumbar spine bone mineral density in AIS, indicating the influence of vitamin D receptor genes [14]. In addition, several factors have been reported to associate with AIS. For example, the composition of the gut microbiome and changes in the plasma proteome correlate with AIS [15]. Age at menarche is reported to be associated with a higher prevalence of AIS, especially in latitudes north of 30 degrees [16]. Moreover, secretion of hormone such as melatonin and estrogen is considered critical in AIS development [17,18]. Despite these findings, the etiopathogenesis of AIS remains controversial.

In this study, we focused on severe AIS patients whose Cobb’s angle is greater than 40 degrees. In such cases, the disease prevalence is relatively low [19] and is more likely to associate with rare genetic variants. Thus, we applied whole-exome sequencing (WES) to investigate susceptibility variants for severe AIS. Rare pathogenic variants within candidate genes were further analyzed by ClinVar database and multiple prediction scores. Moreover, functional annotation databases including knockout mouse phenotypes, Gene Ontology (GO) terms, and KEGG pathways were applied to obtain further insights into the gene function at the biological and physiological levels.

## 2. Materials and Methods

### 2.1. Study Subjects and Sample Preparation

The inclusion criteria for AIS patients in our study were curves likely to reach 45–50 degrees at maturity or curves of 40 degrees in a growing adolescent. The exclusion criteria included patients diagnosed with congenital scoliosis and patients with history of cerebral palsy, Duchenne muscular dystrophy, myelomeningocele, spinal muscular atrophy, or Friedrich ataxia (possibly neuromuscular scoliosis). Accordingly, eleven unrelated AIS patients with a Cobb’s angle greater than 40 degrees were diagnosed and recruited by accredited physicians at Taipei Veterans General Hospital. Clinical data including the Cobb’s angle, AIS classification, and surgical intervention were provided by referring physicians. Peripheral blood samples were collected and transferred to Taipei Medical University for DNA extraction with either phenol-chloroform method or QIAamp Blood Maxi Kit (QIAGEN, Germantown, MD, USA). All subjects gave written informed consent to participate in the study before initiation. This study was approved by the Institutional Review Board (IRB) of Taipei Veterans General Hospital (105DHA0100630).

### 2.2. WES and Bioinformatic Analysis

**Figure 1:**
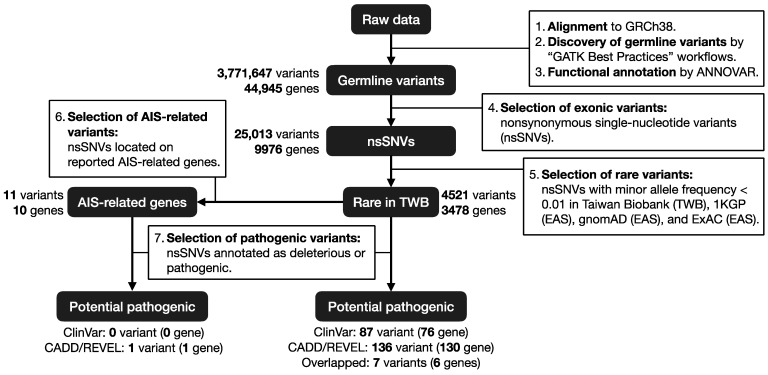
Workflow of the AIS WES analysis. Sequencing reads were aligned to the GRCh38 reference genome and variants were called using GATK Best Practices workflows. Nonsynonymous single-nucleotide variants with minor allele frequency <0.01 were selected. Genes include rare variants were compared with previous AIS-related genes. Rare variants annotated as pathogenic were selected for further analysis.

DNA samples were amplified by PCR and whole-exome library preparation was performed following Nextera WES protocols. Paired-end sequencing (2 × 150 bp) was performed on an Illumina HiSeq 4000, with a sequencing depth above 50× (6 G). Sequencing reads were analyzed through GATK (v4.1.4.0) germline short variant discovery pipeline. In brief, read trimming was performed using Trimmomatic under PE module (ILLUMINACLIP: TruSeq3-PE-2.fa: 2:30:10 LEADING:3 TRAILING:3 SLIDINGWINDOW:4:15 MINLEN:36) to remove adaptors and low-quality bases. Trimmed reads were aligned to the human reference genome GRCh38 using Burrows–Wheeler Aligner (BWA 0.7.12) with default parameters. Samtools were utilized for post-alignment sorting and indexing, followed by PCR duplicates elimination and base quality score recalibration using GATK toolkits. Nonsynonymous single-nucleotide variants were called by GATK HaplotypeCaller. Finally, information of variants was annotated with ANNOVAR (v.20191024). Consequences of the nsSNVs were annotated. Furthermore, to focus on rare variants, we selected variants with minor allele frequency <0.01 in Taiwan Biobank [20], 1000 Genomes Project [21] (1KGP; East Asian), The Genome Aggregation Database [22] (gnomAD; East Asian), and The Exome Aggregation Consortium [23] (ExAC; East Asian). Pathogenicity was further defined by ClinVar annotations and functional prediction scores: (A) variants reported to be either (1) association, (2) conflicting interpretations of pathogenicity, (3) likely pathogenic, (4) pathogenic, or (5) pathogenic/likely pathogenic in ClinVar (v.20210501); (B) variants with CADD Phred scores >20, indicating that the variant is in 1% of the most pathogenic variants; and REVEL raw scores ≥0.75, predicted as pathogenic variants with high specificity but low sensitivity. The study workflow is illustrated in Figure 1.

### 2.3. AIS Related Genes

To identify AIS-related genes, we conducted a gene-based PubMed literature review. Our search keywords were “adolescent idiopathic scoliosis”, “scoliosis”, “gene”, “variants”, “mutations”, and “association” (accessed date at 15 September 2021, https://pubmed.ncbi.nlm.nih.gov). We included genes that are reported to be significantly associated with the onset of AIS, AIS severity, or AIS susceptibility. Therefore, we recruited 45 studies and listed 56 AIS-related genes (Supplementary Table S1).

### 2.4. Functional Annotation

Genes that include pathogenic rare variants and AIS-related genes identified in the sequencing data were selected as candidate genes. In order to gain mechanistic insights into the candidate genes, three gene-based annotations were conducted by using WebGestalt 2019 functional enrichment analysis web tool [24]. Firstly, genes were converted to mouse gene names using BioMart [25] before knockout mouse phenotyping. We used the Mammalian Phenotype Ontology database [26] to observe phenotypic abnormalities in mice with certain genes knocked out. This helps to explain what happens in a human organism when a particular gene is absent. Secondly, we performed a molecular pathway analysis using the Kyoto Encyclopedia of Genes and Genomes (KEGG) [27] to determine how candidate genes are networked and enriched within the pathways. Thirdly, Gene Ontology (GO) [28,29] terms derived from molecular function, cellular component, and biological process were annotated to decipher protein–protein interactions. Only non-redundant categories were contained by selecting the most general categories in each branch of the GO DAG structure. The significance of an enrichment result was set at *q*-value (FDR) < 0.05 for all functional annotations.

## 3. Results

### 3.1. Patient Basal Characteristic

**Figure 2:**
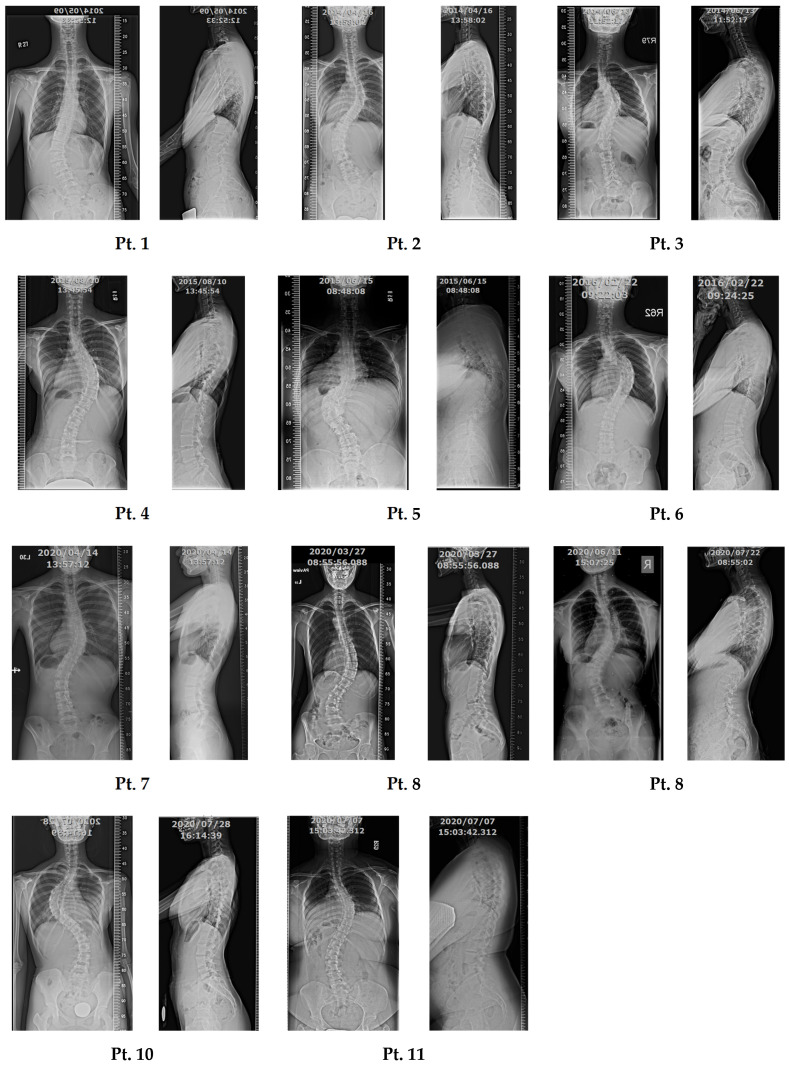
X-rays of 11 AIS patients. Front- and side-view X-rays of each patient were recorded before surgery. The X-ray is used to determine the severity of scoliosis.

We recruited 11 unrelated AIS patients between 14 and 25 years old, including three males and eight females (Table 1). Front- and side-view X-rays were recorded before surgery (Figure 2). All patients had scoliosis with Cobb’s angle greater than 40 degrees.

### 3.2. Pathogenic Rare Variants in Previously Reported AIS-Related Genes

In this study, we firstly collected the published AIS-related genes (Supplementary Table S1). Then, we compared the sequencing data with the AIS-related genes. As shown in Table 2, 11 variants in AIS-related genes were identified in our AIS cohort: *ARF1*, *MAGI1*, *TNIK*, *GC*, *FBN2*, *NT5DC1*, *IL6*, *CSMD1*, *PAX1*, and *TBX1*. A variant *FBN2*(NM_001999):c.809G>T(p.Arg270Leu) was predicted as pathogenic by CADD and REVEL. This variant has not been reported to associate with AIS yet.

### 3.3. Pathogenic Rare Variants on Candidate AIS Genes

**Figure 3:**
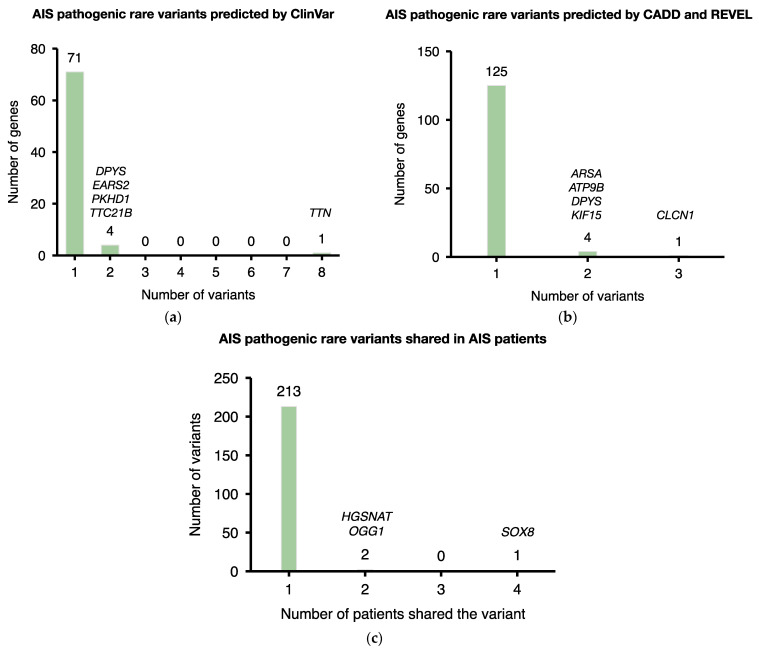
Summary of the pathogenic rare variants identified in 11 severe AIS patients. (**a**) AIS pathogenic rare variants predicted by ClinVar. (**b**) AIS pathogenic rare variants predicted by CADD and REVEL. (**c**) AIS pathogenic rare variants shared in AIS patients.

To prioritize the most important genes in the AIS cohort, we determined the number of pathogenic variants for each candidate genes. Among all 87 pathogenic variants on 76 genes predicted by the ClinVar database, 8 variants were located on *TTN* (Figure 3a, Supplementary Table S2). As for the CADD and REVEL scoring tools, a total of 136 variants on 130 genes were predicted to be pathogenic. Most genes carried only one pathogenic variant, whereas *CLCN1* included three (Figure 3b, Supplementary Table S3). Moreover, among all 216 pathogenic variants predicted by ClinVar database or the CADD/REVEL scores, 213 variants were only carried by one patient in this AIS cohort (Figure 3c). It is noteworthy that *SOX8*(NM_014587):c.685A>C(p.Thr229Pro) was shared by four patients. The *HGSNAT*(NM_001363227):c.205G>A(p.Val69Ile) as well as *OGG1*(NM_001354648):c.461G>A(p.Arg154His) were each carried by two patients.

Importantly, seven variants were identified as pathogenic by both ClinVar database and CADD /REVEL scores (Table 3). These genes include *SGCD*, *CLCN1*, *DPYS*, *MYBPC3*, *MYO7A*, and *NDUFAF5*. Some of these genes were related to muscle functions, of which *SGCD* encodes delta-sarcoglycan and participates in linking the cytoskeleton and the extracellular matrix. *CLCN1* contributes to membrane repolarization in skeletal muscle cells after muscle contraction, and its mutation was known to cause myotonia [30]. It is noteworthy that each variant was carried by a single patient (Table 3, patients 3, 5, 8, 9, and 11). Among them, patient 9 carried three pathogenic variants on *DPYS*, *MYBPC3,* and *MYO7A* respectively. The result implied severe AIS as a polygenic disease, influenced by various genomic loci.

### 3.4. Pathogenic rare Variants Carried by Patient 7

**Figure 4:**
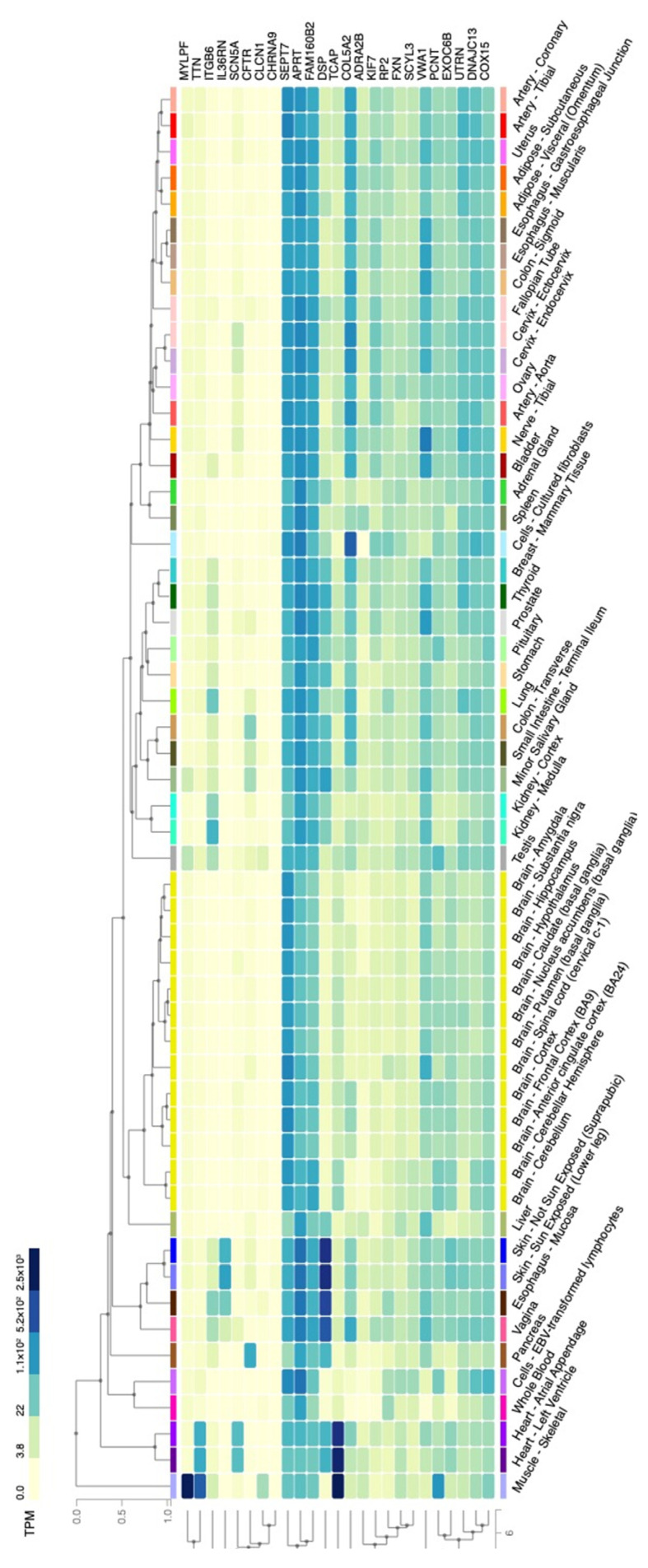
Tissue expression of candidate genes identified in patient 7. The value of gene expression was shown as TPM (Transcript Per Million) and colored according to the color scale at the top left. Each column represents a tissue type. Each row represents the expression level of a certain gene.

In this study, 7 potential variants from both ClinVar and CADD/REVEL and 11 variants within the previous AIS-reported genes are our targets. Although most variants were found in this cohort, none of the pathogenic variants was identified in patient 7. We further carefully screen the results by ClinVar or CADD/REVEL in patient 7, 25 rare variants were proposed (Supplementary Table S4). By querying GTEx, we found that *MYLPF*, *TTN*, and *TCAP* were highly expressed in skeletal muscle (Figure 4). We also highlighted *COL5A2* because *COL5A2* has been implied to associate with AIS [12]. To identify genetic pathology for patient 7, more investigations are still required.

### 3.5. Functional Annotation of AIS Candidate Genes

A total of 206 “AIS candidate genes” were identified according to the selection criteria in the method (Supplementary Table S5). To understand the biological functions, we performed an over-representation analysis (ORA) based on the Mammalian Phenotype Ontology database to determine the impact of candidate genes in mouse phenotypes. A total of 206 human gene names were converted to 200 mouse gene names for further analysis (Supplementary Table S5). In this approach, 102 knockout mouse phenotypes were significantly enriched (Supplementary Table S6). Most phenotypes were related to muscular diseases and the sixth phenotype was abnormal spine curvature. Enriched molecular pathways were examined using KEGG. Four pathways were significantly enriched, and the top three categories were associated with cardiomyopathy, including “hypertrophic cardiomyopathy”, “arrhythmogenic right ventricular”, and “dilated cardiomyopathy” (Table 4).

Furthermore, GO annotations were used to evaluate protein–protein interactions. A total of 60 GO terms were significantly enriched (Supplementary Table S7). Results revealed that a majority of candidate genes involve in cellular movement and muscle contraction, including actin-filament-based movement, contractile fiber, and actin binding terms. We also observed GO terms related to the skeletal system, such as skeletal system morphogenesis and bone development. In addition, several terms are related to the muscle system; for instance, muscle system process, muscle organ development, muscle tissue development, and muscle cell differentiation. Results support the roles of these genes in bone development and muscle function.

## 4. Discussion

Here, we performed whole-exome sequencing on 11 severe AIS patients. Among 200 variants, 9 were located on *TTN*, including 8 variants predicted by ClinVar and 1 by the CADD/REVEL. Three pathogenic variants were found in *CLCN1*. One was predicted by both CADD/REVEL and ClinVar and two were predicted by CADD/REVEL (Supplementary Table S8).

The mutations of *TTN* have been linked to a wide range of dominantly and recessively inherited myopathies [31,32]. Titin is a large protein in the body with scaffold and signaling functions. A previous study revealed *TTN* missense and truncating variants associate with phenotypes of congenital myopathy patients. TTN-related congenital myopathy typically presents clinical features such as scoliosis and respiratory symptoms compare to other myopathies [33]. Nine *TTN* variants we identified were each carried by a single patient. These variants were predicted to affect the function and structure of TTN by either evidence-based databases or prediction-based scoring tools, which suggest the crucial role of *TTN* in the pathogenesis of AIS.

*CLCN1* encodes the chloride voltage-gated channels in the skeletal muscle. *CLCN1* mutations are reported to associate with myotonia congenita [34] and spine phenotypes [35,36]. *Clcn1* knockout mice develop spine deformity, myotonia, muscle weakness, reduced growth, and brittle bones [37]. In this study, we found that CLCN1(NM_000083):c.763G>A(p.Gly255Arg) was carried by patient 7. This missense variant was predicted to be deleterious by the CADD and REVEL scoring tools. The CLCN1(NM_000083):c.892G>A(p.Ala298Thr) and CLCN1(NM_000083):c.1130G>A(p.Arg377Gln) rare variants were observed in patient 11, the former was reported to have a damaging effect on CLCN1 in congenital myotonia patients according to the ClinVar database; the latter was predicted to be deleterious by the CADD and REVEL scoring tools.

*SOX8* is a group E Sox protein closely related to SOX9 and SOX10 that regulates the oligodendroglial specification in the spinal cord [38]. Abnormalities in oligodendrocytes could lead to a variety of neuromuscular diseases, including spinal muscular atrophy (SMA) [39], amyotrophic lateral sclerosis [40], and Duchenne muscular dystrophy (DMD) [41]. It may also contribute to ATR-16 syndrome characterized by alpha-thalassemia and neurodevelopmental disorders [42]. Moreover, SOX8 is associated with myogenic differentiation. Overexpression of SOX8 leads to undifferentiated myoblasts and inability to form myotubes [43]. In this AIS cohort, a pathogenic variant SOX8(NM_014587)c.685A>C(p.Thr229Pro) was carried by four patients (Figure 3c), which was the only variant shared in >35% patients. The results imply *SOX8* as an important susceptibility gene of severe AIS.

This study has several limitations. For example, most previous AIS genetic studies were conducted by GWASs, which mainly focused on common variants. Here, we focus on the rare variants. Thus, the variants we identified are very likely not in the candidate genes by GWAS. Moreover, only variants with a minor allele frequency (MAF) below 0.01 of the East Asian population in multiple databases were selected for the analysis; variants with higher frequencies were removed from this study. Finally, a sample size of 11 individuals in this cohort was not sufficient to comprehensively evaluate the role of candidate genes. In this study, we particularly focused on the AIS patients with a Cobb’s angle greater than 40 degrees. In such severe cases, clinical samples are not as accessible as the common AIS. In addition, collections for family cohorts will be very helpful to investigate the inheritance pattern of critical AIS.

## 5. Conclusions

Our results highlighted *TTN*, *CLCN1*, and *SOX8* as susceptibility genes for severe AIS in a Taiwanese population.

## Figures and Tables

**Table 1:** Basal characteristic of AIS patients.

Sample	Sex	Age [Year]	Location of the Curve	Cobb Angle [Degree]
Pt. 1	M	18	T5-T10	50
Pt. 2	F	17	T10-L3	47
Pt. 3	F	16	T2-T12	45
Pt. 4	F	22	T7-L2	61
Pt. 5	M	25	T9-L2	82
Pt. 6	M	17	T6-T12	72
Pt. 7	F	19	T12-L4/ T11-T6	44/45
Pt. 8	F	24	T12-L4	45
Pt. 9	F	14	T4-T10/T11-L4/	69/66
Pt. 10	F	16	T1-T4/T4-T12/L1-L4	49/86/56
Pt. 11	F	21	T5-T12	50

**Table 2:** Rare variants in AIS-related genes.

Chr.	Position	Allele		Gene	Type	Number of Alternative Alleles in 11 AIS Patients										
		Ref.	Alt.			1	2	3	4	5	6	7	8	9	10	11
1	228097412	G	T	*ARF1*	missense	-	-	1	-	-	-	-	-	-	-	-
3	65478781	G	A	*MAGI1*	missense	-	-	-	1	-	-	-	-	-	-	-
3	171093913	G	A	*TNIK*	missense	-	-	-	-	-	-	-	-	-	-	1
4	71765499	A	T	*GC*	missense	-	-	-	-	-	-	-	-	-	1	-
**5**	**128464741**	**C**	**A**	***FBN2***	**missense**	**1**	**-**	**-**	**-**	**-**	**-**	**-**	**-**	**-**	**-**	**-**
6	116117932	G	A	*NT5DC1*	missense	-	-	-	-	-	1	-	-	-	-	-
7	22727278	A	G	*IL6*	missense	1	-	-	-	-	-	-	-	-	-	-
8	3142671	G	A	*CSMD1*	missense	-	-	-	1	-	-	-	-	-	-	-
20	21709343	C	A	*PAX1*	missense	-	-	-	-	-	-	-	1	-	-	-
22	19761061	C	A	*TBX1*	missense	-	-	-	-	1	-	-	-	-	-	-
22	19761069	C	T	*TBX1*	missense	-	1	-	-	-	-	-	-	-	-	-

All coordinates are from human reference genome GRCh38. Chr.: chromosome. Ref.: reference allele. Alt.: alternative allele. Pathogenic variants are shown in bold.

**Table 3:** AIS pathogenic rare variants predicted by both ClinVar database and the CADD/REVEL scores.

Chr.	Position	Ref.	Alt.	Gene	Type	Allelic Frequency				Pathogenicity			AIS ^a^
						1KGP	gnomAD	ExAC	TWB	ClinVar	CADD	REVEL	
						(*EAS*)	(*EAS*)	(*EAS*)					
5	156759365	A	G	SGCD	missense	-	0.008	0.0064	0.01	Conflicting interpretations of pathogenicity	20.3	0.865	5
7	143330810	G	A	CLCN1	missense	-	-	0.0002	-	Pathogenic/likely pathogenic	29.5	0.9	11
8	104428071	T	C	DPYS	missense	0.003	0.001	0.0013	0.001	Pathogenic	28.1	0.946	8
8	104429590	C	T	DPYS	missense	-	-	0.0001	-	Likely pathogenic	35	0.894	9
11	47346297	C	T	MYBPC3	missense	0.004	0.0026	0.0045	0.001	Conflicting interpretations of pathogenicity	24.9	0.757	9
11	77189442	G	C	MYO7A	missense	0.004	0.0057	0.0042	0.0085	Conflicting interpretations of pathogenicity	28.5	0.853	9
20	13816520	T	G	NDUFAF5	missense	-	0.001	0.0006	0.0015	Conflicting interpretations of pathogenicity	27.7	0.887	3

All coordinates are from human reference genome GRCh38. Chr.: chromosome. Ref.: reference allele. Alt.: alternative allele. ^a^ Patients who carry the variant, each patient carries one copy of the mutation (Heterozygous).

**Table 4:** KEGG pathways enriched in AIS candidate genes.

Pathway Category	No. of Reference Genes	No. of Overlap with AIS Candidate Genes	*p* Value	*q* Value (FDR)
Hypertrophic cardiomyopathy	83	9	3.61 × 10^−6^	1.18 × 10^−3^
Arrhythmogenic right ventricular cardiomyopathy	72	8	1.07 × 10^−5^	1.75 × 10^−3^
Dilated cardiomyopathy	90	8	5.55 × 10^−5^	6.03 × 10^−3^
Lysosome	123	8	4.90 × 10^−4^	3.99 × 10^−2^

## Data Availability

The data presented in this study are available on request from the corresponding author.

## Supplementary Materials

The following supporting information can be downloaded at: https://www.mdpi.com/article/10.3390/jpm13010032/s1, Table S1: AIS-related genes; Table S2: AIS pathogenic rare variants predicted by ClinVar; Table S3: AIS pathogenic rare variants predicted by CADD and REVEL; Table S4: Rare variants carried by patient 7; Table S5: AIS candidate genes; Table S6: Knockout mouse phenotypes enriched in AIS candidate genes; Table S7: GO terms enriched in AIS candidate genes; Table S8: Pathogenic rare variants on *TTN* and *CLCN1*.
