# Supplementary material for: Whole-Exome Sequencing Identifies Genetic Variants for Severe Adolescent Idiopathic Scoliosis in a Taiwanese Population
Source: J Pers Med. 2022 Dec 23;13(1):32. doi: 10.3390/jpm13010032 (PMC9865588; doi:10.3390/jpm13010032)
Supplement: Supplementary file 1 [file jpm-13-00032-s001.zip › jpm-2081226-supplementary.pdf]

Supplementary Table S1. AIS-related genes

No	Gene Name	Location	Exons	Description	Reference
1	AANAT	17q25.1	8	Aralkylamine N-acetyltransferase	[1]
2	ABO	9q34.2	7	Alpha 1-3-N-Acetylgalactosaminyltransferase And Alpha 1-3-Galactosyltransferase	[2]
3	AGMO	7p21.2	21	Mesenchyme homeobox 2	[3]
4	BCKDHB	6q14.1	21	Branched chain keto acid dehydrogenase E1 subunit beta	[3]
5	AJAP1	1p36.32	8	Adherens junctions associated protein 1	[4]
6	AKAP2	9q31.2-q34.2	5	A-kinase anchoring protein 2	[5]
7	ARF1	1q42.13	6	ADP ribosylation factor 1	[3]
8	FAM46A (TENT5A)	6q14.1	3	Terminal nucleotidyltransferase 5A	[3]
9	BCL2	18q21.33	6	BCL2 apoptosis regulator	[4]
10	BNC2	9p22.3-p22.2	9	Basonuclin 2	[6]
11	CALM2	2p21	8	Calmodulin 2	[7]
12	CHD7	8q12.2	42	Chromodomain helicase DNA binding protein 7	[8]
13	CHD13	16q23.3	20	Cadherin 13	[2]
14	CHL1	3p26.3	33	Cell adhesion molecule L1 like	[9]
15	CREB5	7p15.1	19	cAMP responsive element binding protein 5	[3]
16	CSMD1	8p23.2	73	CUB and Sushi multiple domains 1	[3]
17	DPP9	19p13.3	32	Dipeptidyl peptidase 9	[10]
18	EPHA4	2q36.1	19	EPH receptor A4	[4]
19	ESR1	6q25.1-q25.2	23	Estrogen receptor 1	[7, 11]
20	FBN1	15q21.1	66	Fibrillin 1	[12, 13]
21	FBN2	5q23.3	65	Fibrillin 2	[12]
22	FTO	16q12.2	16	FTO alpha-ketoglutarate dependent dioxygenase	[3]
23	GPR126 (ADGRG6)	6q24.2	28	Adhesion G protein-coupled receptor G6	[14]
24	IGF1	12q23.2	7	Insulin like growth factor 1	[15]
25	IL17RC	3p25.3; 3p25.3-p24.1	19	Interleukin 17 receptor C	[16, 17]
26	IL6	7p15.3	6	Interleukin 6	[18]
27	KIF24	9p13.3	17	Kinesin family member 24	[3]
28	LBX1	10q24.32	2	Ladybird homeobox 1	[19, 20]
29	LEPR	1p31.3	24	Leptin receptor	[21]
30	MAGI1	3p14.1	25	Membrane associated guanylate kinase, WW and PDZ domain containing 1	[22]
31	MAPK7	17p11.2	9	Mitogen-activated protein kinase 7	[23, 24]
32	MATN1	1p35.2	8	Matrilin 1	[25, 26]
33	MEIS1	2p14	13	Meis homeobox 1	[22]
34	MEOX2	7p21.2	3	Alkylglycerol monooxygenase	[3]
35	MMP3	11q22.2	10	Matrix metallopeptidase 3	[27]
36	MTMR11	1q21.2	17	Myotubularin related protein 11	[3]
37	MTNR1B	11q14.3	4	Melatonin receptor 1B	[28]
38	NT5DC1	6q22.1	13	5'-nucleotidase domain containing 1	[3]
39	NUCKS1	1q32.1	7	Nuclear casein kinase and cyclin dependent kinase substrate 1	[29]
40	PAX1	20p11.22	5	Paired box 1	[30, 31]
41	PAX3	2q36.1	10	Paired box 3	[4, 32]
42	PLXNA2	1q32.2	34	Plexin A2	[3]
43	POC5	5q13.3	16	POC5 centriolar protein	[33, 34]
44	RUNX2	6p21.1	10	RUNX family transcription factor 2	[35]
45	SH3GL1	19p13.3	12	SH3 domain containing GRB2 like 1, endophilin A2	[36, 37]
46	SLC39A8	4q24	16	Solute carrier family 39 member 8	[38]
47	SOCS3	17q25.3	4	Suppressor of cytokine signaling 3	[39]
48	SOX6	11p15.2	22	SRY-box transcription factor 6	[2]
49	TBX1	22q11.21	13	T-box transcription factor 1	[3]
50	TGFB1	19q13.2	7	Transforming growth factor beta 1	[40]
51	TIMP2	17q25.3	5	TIMP metallopeptidase inhibitor 2	[41, 42]
52	TNIK	3q26.2-q26.31	34	TRAF2 and NCK interacting kinase	[22]
53	TPH1	11p15.1	11	Tryptophan hydroxylase 1	[1]
54	UNCX	7p22.3	3	UNC homeobox	[3]
55	VDBP (GC)	4q13.3	15	GC vitamin D binding protein	[43]
56	VDR	12q13.11	12	Vitamin D receptor	[43-45]

Supplementary Table S2. AIS pathogenic rare variants predicted by ClinVar

Chr	Position	Ref.	Alt.	Gene	Type	Pathogenicity		
						ClinVar	CADD	REVEL
1	2406500	G	A	PEX10	missense	Conflicting interpretations of pathogenicity	24.5	0.115
1	6472613	G	A	PLEKHG5	missense	Conflicting interpretations of pathogenicity	29.9	0.365
1	43427569	G	A	SZT2	missense	Conflicting interpretations of pathogenicity	20.2	0.049
1	77926863	C	T	NEXN	missense	Conflicting interpretations of pathogenicity	32	0.211
1	102879759	C	T	COL11A1	missense	Conflicting interpretations of pathogenicity	24.5	0.446
1	156175609	G	A	SEMA4A	missense	Conflicting interpretations of pathogenicity	4.397	0.015
1	226882007	G	A	PSEN2	missense	Conflicting interpretations of pathogenicity	18.56	0.331
2	26479605	C	T	OTOF	missense	Conflicting interpretations of pathogenicity	13.61	0.203
2	27498276	G	A	GCKR	missense	Conflicting interpretations of pathogenicity	23	0.689
2	113062235	C	T	IL36RN	missense	Conflicting interpretations of pathogenicity	26.3	0.582
2	151562702	A	G	NEB	missense	Conflicting interpretations of pathogenicity	31	0.491
2	165323388	T	C	SCN2A	missense	Conflicting interpretations of pathogenicity	16.31	0.434
2	165880687	G	A	TTC21B	missense	Conflicting interpretations of pathogenicity	26.5	0.341
2	165932983	T	A	TTC21B	missense	Conflicting interpretations of pathogenicity	25.3	0.255
2	178533203	C	T	TTN	missense	Conflicting interpretations of pathogenicity	13.72	0.097
2	178560481	G	T	TTN	missense	Conflicting interpretations of pathogenicity	18.23	0.669
2	178576090	C	T	TTN	missense	Conflicting interpretations of pathogenicity	20.3	0.189
2	178605029	G	A	TTN	missense	Conflicting interpretations of pathogenicity	23.2	0.401
2	178630312	C	A	TTN	missense	Conflicting interpretations of pathogenicity	24.3	0.172
2	178709772	C	T	TTN	missense	Conflicting interpretations of pathogenicity	24.6	0.24
2	178774941	G	C	TTN	missense	Conflicting interpretations of pathogenicity	15.33	0.208
2	178802248	C	T	TTN	missense	Conflicting interpretations of pathogenicity	24	0.499
2	189051367	G	T	COL5A2	missense	Conflicting interpretations of pathogenicity	21.5	0.291
2	219055221	C	T	IHH	missense	Conflicting interpretations of pathogenicity	15.1	0.336
3	49100222	G	A	QARS	missense	Conflicting interpretations of pathogenicity	32	0.536
3	122262227	G	A	CASR	missense	Conflicting interpretations of pathogenicity	12.99	0.219
4	1002327	C	G	IDUA	missense	Conflicting interpretations of pathogenicity	9.866	0.207
4	121861565	T	A	BBS7	missense	Conflicting interpretations of pathogenicity	21	0.582
5	136055770	C	A	TGFBI	missense	Likely pathogenic	25.7	0.629
5	149981105	G	A	SLC26A2	missense	Conflicting interpretations of pathogenicity	24.4	0.64
5	156759365	A	G	SGCD	missense	Conflicting interpretations of pathogenicity	20.3	0.865

6	7585717	A	C	DSP	missense	Conflicting interpretations of pathogenicity	21.9	0.047
6	51747987	G	C	PKHD1	missense	Conflicting interpretations of pathogenicity	24.3	0.49
6	52054153	A	G	PKHD1	missense	Conflicting interpretations of pathogenicity	21.7	0.476
6	73600377	C	T	SLC17A5	missense	Conflicting interpretations of pathogenicity	17.05	0.053
6	129383155	G	A	LAMA2	missense	Conflicting interpretations of pathogenicity	22.1	0.086
6	152293733	C	T	SYNE1	missense	Conflicting interpretations of pathogenicity	34	0.123
7	5987077	C	T	PMS2	missense	Conflicting interpretations of pathogenicity	10.83	0.176
7	117504290	C	T	CFTR	missense	Conflicting interpretations of pathogenicity	27.3	0.669
7	143330810	G	A	CLCN1	missense	Pathogenic/Likely pathogenic	29.5	0.9
8	43147034	G	A	HGSNAT	missense	Conflicting interpretations of pathogenicity	8.446	0.078
8	99853647	G	A	VPS13B	missense	Conflicting interpretations of pathogenicity	3.283	0.141
8	104428071	T	C	DPYS	missense	Pathogenic	28.1	0.946
8	104429590	C	T	DPYS	missense	Likely pathogenic	35	0.894
8	142879710	A	G	CYP11B1	missense	Conflicting interpretations of pathogenicity	5.624	0.146
8	143921312	C	T	PLEC	missense	Conflicting interpretations of pathogenicity	22.1	0.109
9	69046398	G	A	FXN	missense	Conflicting interpretations of pathogenicity	2.046	0.209
9	97685025	G	C	XPA	missense	Conflicting interpretations of pathogenicity	23.6	0.133
10	98433970	G	A	HPS1	missense	Conflicting interpretations of pathogenicity	-	-
11	1753865	C	T	CTSD	missense	Conflicting interpretations of pathogenicity	0.002	0.069
11	47346297	C	T	MYBPC3	missense	Conflicting interpretations of pathogenicity	24.9	0.757
11	65553238	G	A	LTBP3	missense	Conflicting interpretations of pathogenicity	22.7	0.189
11	77189442	G	C	MYO7A	missense	Conflicting interpretations of pathogenicity	28.5	0.853
12	21882797	C	T	ABCC9	missense	Conflicting interpretations of pathogenicity	21.4	0.371
12	32843271	G	A	PKP2	missense	Conflicting interpretations of pathogenicity	0.035	0.02
12	43768124	A	G	IRAK4	missense	Conflicting interpretations of pathogenicity	0.003	0.187
12	50248679	G	T	LIMA1	missense	association	23.6	0.287
12	120978797	C	T	HNF1A	missense	Conflicting interpretations of pathogenicity	23.7	0.634
13	32354955	T	G	BRCA2	missense	Conflicting interpretations of pathogenicity	21.5	0.133
13	51970609	C	T	ATP7B	missense	Conflicting interpretations of pathogenicity	0.014	0.176
14	67729338	C	G	RDH12	missense	Conflicting interpretations of pathogenicity	32	0.667
15	77030525	G	A	PSTPIP1	missense	Conflicting interpretations of pathogenicity	1.016	0.036
15	89630403	G	A	KIF7	missense	Conflicting interpretations of pathogenicity	34	0.525
16	23535179	C	T	EARS2	missense	Conflicting interpretations of pathogenicity	28.7	0.313
16	23552164	T	C	EARS2	missense	Conflicting interpretations of pathogenicity	23.1	0.23

16	23635273	C	T	PALB2	missense	Conflicting interpretations of pathogenicity	16.53	0.058
16	53696210	C	A	RPGRIP1L	missense	Conflicting interpretations of pathogenicity	27.1	0.538
16	57650313	C	T	ADGRG1	missense	Conflicting interpretations of pathogenicity	0.003	0.097
16	89553940	C	G	SPG7	missense	Conflicting interpretations of pathogenicity	28.6	0.559
17	16972035	C	T	TNFRSF13B	missense	Conflicting interpretations of pathogenicity	3.659	0.401
17	39666063	G	A	TCAP	missense	Conflicting interpretations of pathogenicity	13.42	0.517
17	43093525	A	G	BRCA1	missense	Conflicting interpretations of pathogenicity	0.003	0.778
17	65536321	G	A	AXIN2	missense	Conflicting interpretations of pathogenicity	33	0.581
17	80108767	G	A	GAA	missense	Conflicting interpretations of pathogenicity	1.285	0.245
18	46577783	C	T	LOXHD1	missense	Conflicting interpretations of pathogenicity	24.2	0.327
19	35034076	G	A	SCN1B	missense	Conflicting interpretations of pathogenicity	18.09	0.086
19	36058791	G	T	WDR62	missense	Conflicting interpretations of pathogenicity	29.1	0.385
19	40396983	C	T	PRX	missense	Conflicting interpretations of pathogenicity	17.68	0.199
20	13816520	T	G	NDUFAF5	missense	Conflicting interpretations of pathogenicity	27.7	0.887
20	36905449	C	T	SAMHD1	missense	Conflicting interpretations of pathogenicity	16.25	0.259
21	46121586	C	A	COL6A2	missense	Conflicting interpretations of pathogenicity	22	0.594
21	46349722	A	G	PCNT	missense	Conflicting interpretations of pathogenicity	0.002	0.009
22	31861414	C	T	DEPDC5	missense	Conflicting interpretations of pathogenicity	26.5	0.115
X	32545231	G	C	DMD	missense	Conflicting interpretations of pathogenicity	15.32	0.127
X	46837149	C	T	RP2	missense	Likely pathogenic	5.732	0.243
X	69957121	A	C	EDA	missense	Conflicting interpretations of pathogenicity	19.24	0.563
X	154352801	T	C	FLNA	missense	Conflicting interpretations of pathogenicity	0.075	0.274

Supplementary Table S3. AIS pathogenic rare variants predicted by CADD and REVEL

Chr	Position	Ref.	Alt.	Gene	Type	Pathogenicity		
						ClinVar	CADD	REVEL
1	1437479	T	C	VWA1	missense	-	23.3	0.776
1	2303306	C	T	SKI	missense	Uncertain significance	34	0.764
1	6235844	A	G	ICMT	missense	-	20.6	0.794
1	9262281	A	G	H6PD	missense	-	24.8	0.845
1	26022938	G	C	EXTL1	missense	-	25.9	0.816
1	39292770	A	T	MACF1	missense	-	27.1	0.86
1	48229386	G	A	SLC5A9	missense	-	34	0.952
1	84865873	T	C	LPAR3	missense	-	23.4	0.784
1	119764211	A	G	HMGCS2	missense	Uncertain significance	25.1	0.858
1	169876078	T	C	SCYL3	missense	-	24.4	0.962
1	203068501	T	C	PPFIA4	missense	-	26.9	0.916
1	222547756	A	C	HHIPL2	missense	-	24	0.94
2	24017298	C	G	MFSD2B	missense	-	25.2	0.791
2	26279238	C	T	HADHB	missense	-	34	0.889
2	26942037	C	T	DPYSL5	missense	-	31	0.766
2	72514652	T	C	EXOC6B	missense	-	29.6	0.83
2	95410531	T	G	FAHD2A	missense	-	24.1	0.854
2	96114883	G	A	ADRA2B	missense	-	26.4	0.866
2	127574021	A	C	MYO7B	missense	-	26.7	0.763
2	127640126	C	A	LIMS2	missense	-	26.9	0.773
2	151650373	A	T	NEB	missense	-	24.2	0.94
2	160137499	A	G	ITGB6	missense	-	25.8	0.852
2	166199135	C	T	SCN9A	missense	Uncertain significance	34	0.765
2	178738312	C	T	TTN	missense	Likely benign	22.6	0.814
2	189051399	C	T	COL5A2	missense	Benign/Likely benign	27	0.914
2	231037666	T	C	C2orf72	missense	-	23.6	0.77
2	240509382	G	A	ANKMY1	missense	-	32	0.758
3	9751845	G	A	OGG1	missense	-	34	0.938
3	38556545	A	G	SCN5A	missense	Uncertain significance	28.1	0.94
3	44786447	A	G	KIF15	missense	-	26.4	0.836
3	44797927	G	T	KIF15	missense	-	29.3	0.865
3	52766345	C	A	NEK4	missense	-	31	0.95
3	89429165	C	T	EPHA3	missense	-	35	0.774
3	126007159	C	G	SLC41A3	missense	-	29.9	0.778
3	132478076	G	T	DNAJC13	missense	-	21.8	0.762
3	184038163	T	C	HTR3D	missense	-	24.4	0.781
4	24800051	C	T	SOD3	missense	-	32	0.769
4	40335902	G	A	NA9	missense	-	32	0.838
4	54736553	C	T	KIT	missense	Uncertain significance	31	0.889
4	145064603	C	A	ANAPC10	missense	-	33	0.951

4	150849453	A	C	LRBA	missense	-	28.5	0.837
4	151291118	G	T	PRSS48	missense	-	27	0.851
4	169992054	G	A	MFAP3L	missense	-	24.7	0.75
5	60890933	G	C	ERCC8	missense	-	29.6	0.949
5	115837653	A	T	ATG12	missense	-	28.1	0.842
5	128464741	C	A	FBN2	missense	-	29.8	0.824
5	151822699	G	A	GLRA1	missense	Uncertain significance	33	0.786
5	156759365	A	G	SGCD	missense	Conflicting interpretations of pathogenicity	20.3	0.865
5	177397831	T	G	SLC34A1	missense	-	27.6	0.882
6	64626235	C	T	EYS	missense	Uncertain significance	29.6	0.788
6	107459005	A	G	PDSS2	missense	-	33	0.791
6	130890408	G	A	EPB41L2	missense	-	35	0.984
6	144835791	A	T	UTRN	missense	-	29.2	0.837
7	35873733	G	T	SEPTIN7	missense	-	31	0.846
7	41978655	A	T	GLI3	missense	-	28.7	0.923
7	73831563	C	T	CLDN4	missense	-	28.8	0.802
7	92504872	A	G	PEX1	missense	-	24.3	0.817
7	94423084	G	A	COL1A2	missense	-	25.2	0.967
7	99011095	C	T	TRRAP	missense	-	35	0.885
7	143323375	G	A	CLCN1	missense	Uncertain significance	32	0.843
7	143330810	G	A	CLCN1	missense	Pathogenic/Likely pathogenic	29.5	0.9
7	143331616	G	A	CLCN1	missense	-	35	0.915
8	10538492	C	A	PRSS55	missense	-	27.5	0.811
8	11300072	G	T	MTMR9	missense	-	34	0.836
8	17373174	T	C	MTMR7	missense	-	26.1	0.915
8	22100664	T	C	FAM160B2	missense	-	27.5	0.766
8	38234508	G	A	DDHD2	missense	Likely benign	34	0.75
8	104428071	T	C	DPYS	missense	Pathogenic	28.1	0.946
8	104429590	C	T	DPYS	missense	Likely pathogenic	35	0.894
8	138676616	C	A	COL22A1	missense	-	23.3	0.832
8	143839988	G	T	NRBP2	missense	-	26.1	0.76
9	39078875	C	T	CNTNAP3	missense	-	25	0.763
9	100578538	T	A	CAVIN4	missense	-	34	0.883
9	109255578	G	A	EPB41L4B	missense	-	34	0.761
10	33213531	C	T	NRP1	missense	-	26.2	0.75
10	69005904	T	C	KIF1BP	missense	-	28.3	0.825
10	69483695	T	C	TSPAN15	missense	-	25.4	0.768
10	70142673	A	T	TYSND1	missense	-	24.1	0.782
10	99716418	G	C	COX15	missense	-	27.2	0.835
11	6240139	C	A	CNGA4	missense	-	23.6	0.759
11	6614985	T	G	TPP1	missense	-	27	0.929
11	47346297	C	T	MYBPC3	missense	Conflicting interpretations of pathogenicity	24.9	0.757
11	68348146	C	T	LRP5	missense	-	31	0.869

11	77189442	G	C	MYO7A	missense	Conflicting interpretations of pathogenicity	28.5	0.853
11	112194746	G	A	BCO2	missense	-	26.1	0.862
12	2611975	G	A	CACNA1C	missense	Uncertain significance	25.1	0.779
12	21642074	C	T	LDHB	missense	-	24.5	0.844
12	49963585	C	T	AQP5	missense	-	33	0.762
12	52601034	A	T	KRT72	missense	-	32	0.953
12	121854776	C	T	HPD	missense	-	32	0.863
13	24284248	G	A	SPATA13	missense	-	33	0.764
13	38968452	A	G	STOML3	missense	-	26.8	0.927
13	76995986	G	C	CLN5	missense	Uncertain significance	25.5	0.848
14	90676545	C	T	TTC7B	missense	-	33	0.789
15	40411270	G	C	IVD	missense	Uncertain significance	27.6	0.93
15	45094679	G	A	DUOX2	missense	Uncertain significance	35	0.845
15	71991147	G	A	MYO9A	missense	-	33	0.81
15	89750873	A	G	MESP1	missense	-	25.2	0.88
15	98891348	C	T	IGF1R	missense	-	23.6	0.763
16	984730	A	C	SOX8	missense	-	24.5	0.822
16	4260115	C	T	TFAP4	missense	-	29.3	0.76
16	30376199	C	T	MYLPF	missense	-	34	0.765
16	48178620	C	T	ABCC11	missense	-	26	0.816
16	72798598	A	G	ZFHX3	missense	-	22.8	0.796
16	84222725	G	A	KCNG4	missense	-	28.6	0.807
16	88810124	C	T	APRT	missense	Uncertain significance	26.7	0.863
17	1480776	C	A	MYO1C	missense	-	29.4	0.925
17	18959147	C	T	SLC5A10	missense	-	32	0.866
17	21703480	C	T	KCNJ18	missense	-	25.9	0.84
17	29095054	T	C	MYO18A	missense	-	31	0.893
17	39107505	A	G	PLXDC1	missense	-	26.6	0.876
17	41884269	G	A	ACLY	missense	-	31	0.764
18	48950022	T	C	SMAD7	missense	-	23	0.805
18	79126315	C	T	ATP9B	missense	-	34	0.816
18	79277071	A	T	ATP9B	missense	-	31	0.899
19	13235658	A	G	CACNA1A	missense	-	23	0.831
19	33512643	C	T	PEPD	missense	-	33	0.905
19	40502809	G	A	SPTBN4	missense	-	34	0.75
19	48836366	C	T	HSD17B14	missense	-	34	0.752
19	50445880	G	C	MYBPC2	missense	-	23.8	0.85
20	6085278	A	G	FERMT1	missense	-	31	0.94
20	13816520	T	G	NDUFAF5	missense	Conflicting interpretations of pathogenicity	27.7	0.887
20	23048217	C	T	THBD	missense	-	29.4	0.814
20	25259273	C	T	PYGB	missense	-	35	0.936
20	36615277	G	C	SLA2	missense	-	25.5	0.792
20	42081933	G	C	PTPRT	missense	-	28.8	0.778

20	62798639	C	T	MRGBP	missense	-	33	0.794
21	38299527	C	T	KCNJ15	missense	-	24	0.751
21	42121098	T	C	UMODL1	missense	-	24.1	0.871
21	44312232	T	A	PFKL	missense	-	25.4	0.935
22	21968713	C	T	TOP3B	missense	-	33	0.923
22	50576537	C	A	CPT1B	missense	-	32	0.767
22	50625378	G	C	ARSA	missense	Likely benign	22.6	0.75
22	50626061	C	T	ARSA	missense	Likely benign	28	0.788
X	49216406	A	C	CACNA1F	missense	-	23.7	0.974
X	100599568	T	C	TNMD	missense	-	26.4	0.78

Supplementary Table S4. Rare variants carried by patient 7

Chr	Position	Ref	Alt.	Gene	Type	avSNP150	TWB	Pathogenicity	
								ClinVar	CADD/ REVEL
1	1437479	T	C	VWA1	missense	rs753372307	-	-	23.3/0.776
1	169876078	T	C	SCYL3	missense	-	-	-	24.4/0.962
2	113062235	C	T	IL36RN	missense	rs139497891	-	Conflicting interpretations of pathogenicity	26.3/0.582
2	178802248	C	T	TTN	missense	rs758169489	0.0015	Conflicting interpretations of pathogenicity	24/0.499
2	72514652	T	C	EXOC6B	missense	-	-	-	29.6/0.83
2	96114883	G	A	ADRA2B	missense	rs764017593	0.0005	-	26.4/0.866
2	160137499	A	G	ITGB6	missense	rs376026402	-	-	25.8/0.852
2	189051399	C	T	COL5A2	missense	rs772448543	0.005	Benign/Likely benign	27/0.914
3	38556545	A	G	SCN5A	missense	rs754733108	-	Uncertain significance	28.1/0.94
3	132478076	G	T	DNAJC13	missense	rs761450652	0.0015	-	21.8/0.762
4	40335902	G	A	CHRNA9	missense	rs188109471	0.001	-	32/0.838
6	7585717	A	C	DSP	missense	rs138329459	0.0015	Conflicting interpretations of pathogenicity	21.9/0.047
6	144835791	A	T	UTRN	missense	-	-	-	29.2/0.837
7	117504290	C	T	CFTR	missense	rs1800073	0.007	Conflicting interpretations of pathogenicity	27.3/0.669
7	35873733	G	T	SEPTIN7	missense	-	-	-	31/0.846
7	143323375	G	A	CLCN1	missense	rs746691295	-	Uncertain significance	32/0.843
8	22100664	T	C	FAM160B2	missense	rs780699373	-	-	27.5/0.766
9	69046398	G	A	FXN	missense	rs141858334	0.004	Conflicting interpretations of pathogenicity	2.046/0.209
10	99716418	G	C	COX15	missense	-	-	-	27.2/0.835
15	89630403	G	A	KIF7	missense	rs147191956	-	Conflicting interpretations of pathogenicity	34/0.525
16	30376199	C	T	MYLPF	missense	rs368612339	-	-	34/0.765
16	88810124	C	T	APRT	missense	rs201944035	0.003	Uncertain significance	26.7/0.863
17	39666063	G	A	TCAP	missense	rs149585781	-	Conflicting interpretations of pathogenicity	13.42/0.517
21	46349722	A	G	PCNT	missense	rs368199588	0.001	Conflicting interpretations of pathogenicity	0.002/0.009
X	46837149	C	T	RP2	missense	rs782103396	-	Likely pathogenic	5.732/0.243

Supplementary Table S5. AIS candidate genes

Human Associated Gene Name (N=206)	Mouse Associated Gene Name (N=200)	Ensembl gene ID (N=200)	Note
ABCC11	-	-	Can't mapped to mouse gene name
ABCC9	Abcc9	ENSMUSG00000030249	-
ACLY	Acly	ENSMUSG00000020917	-
ADGRG1	Adgrg1	ENSMUSG00000031785	-
ADRA2B	Adra2b	ENSMUSG00000058620	-
ANAPC10	Anapc10	ENSMUSG00000036977	-
ANKMY1	Ankmy1	ENSMUSG00000034212	-
APRT	Aprt	ENSMUSG00000006589	-
AQP5	Aqp5	ENSMUSG00000044217	-
ARF1	Arf1	ENSMUSG00000048076	-
ARSA	Arsa	ENSMUSG00000022620	-
ATG12	Atg12	ENSMUSG00000032905	-
ATP7B	Atp7b	ENSMUSG00000006567	-
ATP9B	Atp9b	ENSMUSG00000024566	-
AXIN2	Axin2	ENSMUSG00000000142	-
BBS7	Bbs7	ENSMUSG00000037325	-
BCO2	Bco2	ENSMUSG00000032066	-
BRCA1	Brca1	ENSMUSG00000017146	-
BRCA2	Brca2	ENSMUSG00000041147	-
C2orf72	2810459M11Rik	ENSMUSG00000026227	-
CACNA1A	Cacna1a	ENSMUSG00000034656	-
CACNA1C	Cacna1c	ENSMUSG00000051331	-
CACNA1F	Cacna1f	ENSMUSG00000031142	-
CASR	Casr	ENSMUSG00000051980	-
CAVIN4	Cavin4	ENSMUSG00000028348	-
CFTR	Cftr	ENSMUSG00000041301	-
CHRNA9	Chrna9	ENSMUSG00000029205	-
CLCN1	Clcn1	ENSMUSG00000029862	-
CLDN4	Cldn13	ENSMUSG00000008843	Mapped to multiple mouse gene name
CLDN4	Cldn4	ENSMUSG00000047501	Mapped to multiple mouse gene name
CLN5	Cln5	ENSMUSG00000022125	-
CNGA4	Cnga4	ENSMUSG00000030897	-
CNTNAP3	Cntnap3	ENSMUSG00000033063	-
COL11A1	Col11a1	ENSMUSG00000027966	-
COL1A2	Col1a2	ENSMUSG00000029661	-
COL22A1	-	-	Can't mapped to mouse gene name
COL5A2	Col5a2	ENSMUSG00000026042	-
COL6A2	Col6a2	ENSMUSG00000020241	-
COX15	Cox15	ENSMUSG00000040018	-
CPT1B	Cpt1b	ENSMUSG00000078937	-
CSMD1	Csmd1	ENSMUSG00000060924	-
CTSD	Gm49369	ENSMUSG00000110040	Mapped to multiple mouse gene name
CTSD	Ctsd	ENSMUSG00000007891	Mapped to multiple mouse gene name
CYP11B1	-	-	Can't mapped to mouse gene name
DDHD2	Ddhd2	ENSMUSG00000061313	-
DEPDC5	Depdc5	ENSMUSG00000037426	-
DMD	Dmd	ENSMUSG00000045103	-
DNAJC13	Dnajc13	ENSMUSG00000032560	-
DPYS	Dpys	ENSMUSG00000022304	-

DPYSL5	Dpysl5	ENSMUSG00000029168	-
DSP	Dsp	ENSMUSG00000054889	-
DUOX2	Duox2	ENSMUSG00000068452	-
EARS2	Ears2	ENSMUSG00000030871	-
EDA	Eda	ENSMUSG00000059327	-
EPB41L2	Epb41l2	ENSMUSG00000019978	-
EPB41L4B	Epb41l4b	ENSMUSG00000028434	-
EPHA3	Epha3	ENSMUSG00000052504	-
ERCC8	Ercc8	ENSMUSG00000021694	-
EXOC6B	Exoc6b	ENSMUSG00000033769	-
EXTL1	Extl1	ENSMUSG00000028838	-
EYS	-	-	Can't mapped to mouse gene name
FAHD2A	Fahd2a	ENSMUSG00000027371	-
FAM160B2	-	-	Can't mapped to mouse gene name
FBN2	Fbn2	ENSMUSG00000024598	-
FERMT1	Fermt1	ENSMUSG00000027356	-
FLNA	Flna	ENSMUSG00000031328	-
FXN	Fxn	ENSMUSG00000059363	-
GAA	Gaa	ENSMUSG00000025579	-
GC	Gc	ENSMUSG00000035540	-
GCKR	Gckr	ENSMUSG00000059434	-
GLI3	Gli3	ENSMUSG00000021318	-
GLRA1	Glra1	ENSMUSG00000000263	-
H6PD	H6pd	ENSMUSG00000028980	-
HADHB	Hadhb	ENSMUSG00000059447	-
HGSNAT	Hgsnat	ENSMUSG00000037260	-
HHIPL2	Hhipl2	ENSMUSG00000053461	-
HMGCS2	Hmgcs2	ENSMUSG00000027875	-
HNF1A	Hnf1a	ENSMUSG00000029556	-
HPD	Hpd	ENSMUSG00000029445	-
HPS1	Hps1	ENSMUSG00000025188	-
HSD17B14	Hsd17b14	ENSMUSG00000030825	-
HTR3D	-	-	Can't mapped to mouse gene name
ICMT	Icmt	ENSMUSG00000039662	-
IDUA	Idua	ENSMUSG00000033540	-
IGF1R	Igf1r	ENSMUSG00000005533	-
IHH	Ihh	ENSMUSG00000006538	-
IL36RN	Il36rn	ENSMUSG00000026983	-
IL6	Il6	ENSMUSG00000025746	-
IRAK4	Irak4	ENSMUSG00000059883	-
ITGB6	Itgb6	ENSMUSG00000026971	-
IVD	Ivd	ENSMUSG00000027332	-
KCNG4	Kcng4	ENSMUSG00000045246	-
KCNJ15	Kcnj15	ENSMUSG00000062609	-
KCNJ18	Kcnj12	ENSMUSG00000042529	-
KIF15	Kif15	ENSMUSG00000036768	-
KIF1BP	-	-	Can't mapped to mouse gene name
KIF7	Kif7	ENSMUSG00000050382	-
KIT	Kit	ENSMUSG00000005672	-
KRT72	Krt72	ENSMUSG00000056605	-
LAMA2	Lama2	ENSMUSG00000019899	-
LDHB	Ldhb	ENSMUSG00000030246	-
LIMA1	Lima1	ENSMUSG00000023022	-

LIMS2	Lims2	ENSMUSG00000024395	-
LOXHD1	Loxhd1	ENSMUSG00000032818	-
LPAR3	Lpar3	ENSMUSG00000036832	-
LRBA	Lrba	ENSMUSG00000028080	-
LRP5	Lrp5	ENSMUSG00000024913	-
LTBP3	Ltbp3	ENSMUSG00000024940	-
MACF1	Macf1	ENSMUSG00000028649	-
MAGI1	Magi1	ENSMUSG00000045095	-
MESP1	Mesp1	ENSMUSG00000030544	-
MFAP3L	Mfap3l	ENSMUSG00000031647	-
MFSD2B	Mfsd2b	ENSMUSG00000037336	-
MRGBP	Mrgbp	ENSMUSG00000027569	-
MTMR7	Mtmr7	ENSMUSG00000039431	-
MTMR9	Mtmr9	ENSMUSG00000035078	-
MYBPC2	Mybpc2	ENSMUSG00000038670	-
MYBPC3	Mybpc3	ENSMUSG00000002100	-
MYLPF	Mylpf	ENSMUSG00000030672	-
MYO18A	Myo18a	ENSMUSG00000000631	-
MYO1C	Myo1c	ENSMUSG00000017774	-
MYO7A	Myo7a	ENSMUSG00000030761	-
MYO7B	Myo7b	ENSMUSG00000024388	-
MYO9A	Myo9a	ENSMUSG00000039585	-
NDUFAF5	Ndufaf5	ENSMUSG00000027384	-
NEB	Neb	ENSMUSG00000026950	-
NEK4	Nek4	ENSMUSG00000021918	-
NEXN	Nexn	ENSMUSG00000039103	-
NRBP2	Nrbp2	ENSMUSG00000075590	-
NRP1	Nrp1	ENSMUSG00000025810	-
NT5DC1	Nt5dc1	ENSMUSG00000039480	-
OGG1	Ogg1	ENSMUSG00000030271	-
OTOF	Otof	ENSMUSG00000062372	-
PALB2	Palb2	ENSMUSG00000044702	-
PAX1	Pax1	ENSMUSG00000037034	-
PCNT	Pcnt	ENSMUSG00000001151	-
PDSS2	Pdss2	ENSMUSG00000038240	-
PEPD	Pepd	ENSMUSG00000063931	-
PEX1	Pex1	ENSMUSG00000005907	-
PEX10	Pex10	ENSMUSG00000029047	-
PFKL	Pfkl	ENSMUSG00000020277	-
PKHD1	Pkhd1	ENSMUSG00000043760	-
PKP2	Pkp2	ENSMUSG00000041957	-
PLEC	Plec	ENSMUSG00000022565	-
PLEKHG5	Plekhg5	ENSMUSG00000039713	-
PLXDC1	Plxdc1	ENSMUSG00000017417	-
PMS2	Pms2	ENSMUSG00000079109	-
PPFIA4	Ppfia4	ENSMUSG00000026458	-
PRSS48	Prss48	ENSMUSG00000049013	-
PRSS55	Prss55	ENSMUSG00000034623	-
PRX	Prx	ENSMUSG00000053198	-
PSEN2	Psen2	ENSMUSG00000010609	-
PSTPIP1	Pstpip1	ENSMUSG00000032322	-
PTPRT	Ptprt	ENSMUSG00000053141	-
PYGB	Pygb	ENSMUSG00000033059	-

QARS	-	-	Can't mapped to mouse gene name
RDH12	Rdh12	ENSMUSG00000021123	-
RP2	Rp2	ENSMUSG00000060090	-
RPGRIP1L	Rpgrip1l	ENSMUSG00000033282	-
SAMHD1	Samhd1	ENSMUSG00000027639	-
SCN1B	Scn1b	ENSMUSG00000019194	-
SCN2A	Scn2a	ENSMUSG00000075318	-
SCN5A	Scn5a	ENSMUSG00000032511	-
SCN9A	Scn9a	ENSMUSG00000075316	-
SCYL3	Scyl3	ENSMUSG00000026584	-
SEMA4A	Sema4a	ENSMUSG00000028064	-
SEPTIN7	Septin7	ENSMUSG00000001833	-
SGCD	Sgcd	ENSMUSG00000020354	-
SKI	Ski	ENSMUSG00000029050	-
SLA2	Sla2	ENSMUSG00000027636	-
SLC17A5	Slc17a5	ENSMUSG00000049624	-
SLC26A2	Slc26a2	ENSMUSG00000034320	-
SLC34A1	Slc34a1	ENSMUSG00000021490	-
SLC41A3	Slc41a3	ENSMUSG00000030089	-
SLC5A10	Slc5a10	ENSMUSG00000042371	-
SLC5A9	Slc5a9	ENSMUSG00000028544	-
SMAD7	Smad7	ENSMUSG00000025880	-
SOD3	Sod3	ENSMUSG00000072941	-
SOX8	Sox8	ENSMUSG00000024176	-
SPATA13	Spata13	ENSMUSG00000021990	-
SPG7	Spg7	ENSMUSG00000000738	-
SPTBN4	Sptbn4	ENSMUSG00000011751	-
STOML3	Stoml3	ENSMUSG00000027744	-
SYNE1	Syne1	ENSMUSG00000096054	-
SZT2	Szt2	ENSMUSG00000033253	-
TBX1	Tbx1	ENSMUSG00000009097	-
TCAP	Tcap	ENSMUSG00000007877	-
TFAP4	Tfap4	ENSMUSG00000005718	-
TGFBI	Tgfbi	ENSMUSG00000035493	-
THBD	Thbd	ENSMUSG00000074743	-
TNFRSF13B	Tnfrsf13b	ENSMUSG00000010142	-
TNIK	Tnik	ENSMUSG00000027692	-
TNMD	Tnmd	ENSMUSG00000031250	-
TOP3B	Top3b	ENSMUSG00000022779	-
TPP1	Tpp1	ENSMUSG00000030894	-
TRRAP	Trrap	ENSMUSG00000045482	-
TSPAN15	Tspan15	ENSMUSG00000037031	-
TTC21B	Ttc21b	ENSMUSG00000034848	-
TTC7B	Ttc7b	ENSMUSG00000033530	-
TTN	Ttn	ENSMUSG00000051747	-
TYSND1	Tysnd1	ENSMUSG00000020087	-
UMODL1	Umodl1	ENSMUSG00000054134	-
UTRN	Utrn	ENSMUSG00000019820	-
VPS13B	Vps13b	ENSMUSG00000037646	-
VWA1	Vwa1	ENSMUSG00000042116	-
WDR62	Wdr62	ENSMUSG00000037020	-
XPA	Xpa	ENSMUSG00000028329	-
ZFHX3	Zfhx3	ENSMUSG00000038872	-

Supplementary Table S6. Knockout mouse phenotypes enriched in AIS candidate genes

Phenotype category	No. of reference genes	No. of overlap with AIS candidate genes	*p*-value	q-value (FDR)
muscle phenotype	1282	48	2.36×10^{-9}	1.54×10^{-5}
abnormal neuron morphology	1243	45	2.56×10^{-8}	8.35×10^{-5}
abnormal muscle morphology	928	36	1.77×10^{-7}	1.99×10^{-4}
decreased body weight	1615	51	1.96×10^{-7}	1.99×10^{-4}
decreased total tissue mass	1615	51	1.96×10^{-7}	1.99×10^{-4}
abnormal spine curvature	242	17	1.97×10^{-7}	1.99×10^{-4}
abnormal muscle physiology	770	32	2.14×10^{-7}	1.99×10^{-4}
premature death	952	36	3.33×10^{-7}	2.71×10^{-4}
abnormal total tissue mass	1814	54	5.15×10^{-7}	3.59×10^{-4}
abnormal muscle fiber morphology	322	19	5.50×10^{-7}	3.59×10^{-4}
abnormal skeletal muscle fiber morphology	169	13	2.11×10^{-6}	1.16×10^{-3}
abnormal muscle electrophysiology	26	6	2.27×10^{-6}	1.16×10^{-3}
postnatal growth retardation	773	30	2.31×10^{-6}	1.16×10^{-3}
abnormal postnatal growth	798	30	4.40×10^{-6}	2.05×10^{-3}
abnormal body weight	1945	54	4.76×10^{-6}	2.07×10^{-3}
myopathy	46	7	6.12×10^{-6}	2.44×10^{-3}
abnormal skeletal muscle morphology	381	19	6.67×10^{-6}	2.44×10^{-3}
abnormal QRS complex	65	8	6.73×10^{-6}	2.44×10^{-3}
kyphosis	162	12	7.80×10^{-6}	2.68×10^{-3}
centrally nucleated skeletal muscle fibers	70	8	1.18×10^{-5}	3.84×10^{-3}
abnormal somatic nervous system morphology	845	30	1.37×10^{-5}	4.25×10^{-3}
abnormal gait	372	18	1.79×10^{-5}	4.82×10^{-3}
dilated heart	176	12	1.81×10^{-5}	4.82×10^{-3}
short stride length	54	7	1.83×10^{-5}	4.82×10^{-3}
cardiac fibrosis	122	10	1.90×10^{-5}	4.82×10^{-3}
abnormal heart electrocardiography waveform feature	149	11	1.95×10^{-5}	4.82×10^{-3}
abnormal locomotor coordination	566	23	1.99×10^{-5}	4.82×10^{-3}
abnormal muscle contractility	315	16	2.99×10^{-5}	6.96×10^{-3}
neurodegeneration	388	18	3.13×10^{-5}	7.04×10^{-3}
respiratory system phenotype	1209	37	3.26×10^{-5}	7.08×10^{-3}
absent vertebral body	5	3	3.59×10^{-5}	7.55×10^{-3}
heart block	61	7	4.11×10^{-5}	8.11×10^{-3}
abnormal stride length	61	7	4.11×10^{-5}	8.11×10^{-3}
decreased cardiac muscle contractility	163	11	4.49×10^{-5}	8.61×10^{-3}
abnormal glial cell morphology	296	15	5.50×10^{-5}	1.03×10^{-2}
abnormal impulse conducting system conduction	169	11	6.24×10^{-5}	1.13×10^{-2}
increased inflammatory response	873	29	6.63×10^{-5}	1.16×10^{-2}
abnormal heart ventricle morphology	612	23	6.76×10^{-5}	1.16×10^{-2}
skeletal muscle necrosis	29	5	7.43×10^{-5}	1.21×10^{-2}
prolonged QRS complex duration	29	5	7.43×10^{-5}	1.21×10^{-2}
abnormal respiratory system physiology	748	26	8.00×10^{-5}	1.26×10^{-2}
vision/eye phenotype	1462	41	8.36×10^{-5}	1.26×10^{-2}
abnormal locomotor behavior	1365	39	8.79×10^{-5}	1.26×10^{-2}
intraventricular block	30	5	8.81×10^{-5}	1.26×10^{-2}
bundle branch block	30	5	8.81×10^{-5}	1.26×10^{-2}

abnormal voluntary movement	1466	41	8.89×10^{-5}	1.26×10^{-2}
abnormal inner ear morphology	276	14	9.73×10^{-5}	1.35×10^{-2}
abnormal myocardial fiber morphology	148	10	9.93×10^{-5}	1.35×10^{-2}
cardiomyopathy	94	8	1.01×10^{-4}	1.35×10^{-2}
dystrophic muscle	31	5	1.04×10^{-4}	1.35×10^{-2}
skeletal muscle fiber necrosis	17	4	1.14×10^{-4}	1.45×10^{-2}
abnormal microglial cell morphology	72	7	1.20×10^{-4}	1.51×10^{-2}
abnormal eye morphology	1346	38	1.43×10^{-4}	1.76×10^{-2}
abnormal somatic sensory system morphology	603	22	1.52×10^{-4}	1.83×10^{-2}
abnormal auditory brainstem response	363	16	1.60×10^{-4}	1.90×10^{-2}
postnatal lethality	1014	31	1.67×10^{-4}	1.95×10^{-2}
abnormal cochlea morphology	222	12	1.72×10^{-4}	1.97×10^{-2}
impaired muscle contractility	224	12	1.87×10^{-4}	2.04×10^{-2}
abnormal retinal photoreceptor morphology	160	10	1.89×10^{-4}	2.04×10^{-2}
abnormal Cajal-Retzius cell morphology	8	3	1.94×10^{-4}	2.04×10^{-2}
abnormal A band morphology	8	3	1.94×10^{-4}	2.04×10^{-2}
abnormal exercise endurance	55	6	1.97×10^{-4}	2.04×10^{-2}
muscle weakness	78	7	1.99×10^{-4}	2.04×10^{-2}
abnormal respiration	489	19	2.00×10^{-4}	2.04×10^{-2}
decreased skeletal muscle fiber size	79	7	2.16×10^{-4}	2.17×10^{-2}
decreased skeletal muscle fiber diameter	37	5	2.47×10^{-4}	2.44×10^{-2}
dilated heart left ventricle	81	7	2.53×10^{-4}	2.46×10^{-2}
abnormal hearing electrophysiology	380	16	2.69×10^{-4}	2.58×10^{-2}
abnormal retinal photoreceptor layer morphology	168	10	2.81×10^{-4}	2.66×10^{-2}
deafness	110	8	3.03×10^{-4}	2.82×10^{-2}
abnormal heart morphology	1297	36	3.14×10^{-4}	2.82×10^{-2}
abnormal skeletal muscle fiber size	111	8	3.22×10^{-4}	2.82×10^{-2}
abnormal basicranium morphology	111	8	3.22×10^{-4}	2.82×10^{-2}
increased or absent threshold for auditory brainstem response	310	14	3.26×10^{-4}	2.82×10^{-2}
abnormal ileum morphology	22	4	3.29×10^{-4}	2.82×10^{-2}
prolonged PR interval	22	4	3.29×10^{-4}	2.82×10^{-2}
abnormal membranous labyrinth morphology	239	12	3.40×10^{-4}	2.88×10^{-2}
hearing/vestibular/ear phenotype	685	23	3.57×10^{-4}	2.97×10^{-2}
abnormal inflammatory response	1059	31	3.62×10^{-4}	2.97×10^{-2}
abnormal cochlear ganglion morphology	86	7	3.66×10^{-4}	2.97×10^{-2}
abnormal ear physiology	472	18	3.72×10^{-4}	2.97×10^{-2}
abnormal scala media morphology	207	11	3.73×10^{-4}	2.97×10^{-2}
abnormal cardiac muscle contractility	208	11	3.89×10^{-4}	3.02×10^{-2}
abnormal cochlear labyrinth morphology	208	11	3.89×10^{-4}	3.02×10^{-2}
decreased aerobic running capacity	10	3	4.06×10^{-4}	3.09×10^{-2}
abnormal cochlear hair cell morphology	176	10	4.08×10^{-4}	3.09×10^{-2}
abnormal hearing physiology	436	17	4.23×10^{-4}	3.17×10^{-2}
abnormal cardiovascular system morphology	1794	45	4.70×10^{-4}	3.48×10^{-2}
impaired exercise endurance	43	5	5.06×10^{-4}	3.69×10^{-2}
abnormal cochlear sensory epithelium morphology	181	10	5.09×10^{-4}	3.69×10^{-2}
abnormal organ of Corti morphology	182	10	5.31×10^{-4}	3.81×10^{-2}

dilated heart ventricle	120	8	5.45×10^{-4}	3.81×10^{-2}
prolonged P wave	11	3	5.53×10^{-4}	3.81×10^{-2}
atrioventricular valve regurgitation	11	3	5.53×10^{-4}	3.81×10^{-2}
abnormal hair cell morphology	183	10	5.55×10^{-4}	3.81×10^{-2}
abnormal skeletal muscle mass	121	8	5.76×10^{-4}	3.92×10^{-2}
abnormal heart layer morphology	368	15	5.91×10^{-4}	3.97×10^{-2}
abnormal brain morphology	1444	38	5.98×10^{-4}	3.98×10^{-2}
thick ventricular wall	45	5	6.26×10^{-4}	4.11×10^{-2}
hindlimb paralysis	68	6	6.29×10^{-4}	4.11×10^{-2}
abnormal axon morphology	157	9	7.45×10^{-4}	4.81×10^{-2}
lethality during fetal growth through weaning	1890	46	7.80×10^{-4}	4.99×10^{-2}

Supplementary Table S7. GO terms enriched in AIS candidate genes

GO term	No. of reference genes	No. of overlap with AIS-genes of interest	*p*-value	q-value (FDR)
actin filament-based movement	132	15	7.50×10^{-11}	9.78×10^{-8}
Sarcolemma	134	14	1.01×10^{-9}	6.59×10^{-7}
structural constituent of muscle	44	9	2.52×10^{-9}	8.64×10^{-7}
contractile fiber	226	17	2.65×10^{-9}	8.64×10^{-7}
actin binding	419	22	9.70×10^{-9}	2.53×10^{-6}
muscle system process	423	21	5.62×10^{-8}	1.22×10^{-5}
muscle organ development	380	19	2.24×10^{-7}	4.18×10^{-5}
heart morphogenesis	250	15	4.58×10^{-7}	7.46×10^{-5}
regulation of membrane potential	414	19	8.26×10^{-7}	1.20×10^{-4}
passive transmembrane transporter activity	457	19	3.55×10^{-6}	4.63×10^{-4}
circulatory system process	480	19	7.18×10^{-6}	8.51×10^{-4}
multicellular organismal signaling	170	11	7.96×10^{-6}	8.65×10^{-4}
sodium ion transport	213	12	1.26×10^{-5}	1.19×10^{-3}
sensory organ morphogenesis	250	13	1.29×10^{-5}	1.19×10^{-3}
muscle tissue development	371	16	1.37×10^{-5}	1.19×10^{-3}
pigmentation	95	8	2.14×10^{-5}	1.75×10^{-3}
regulation of ion transmembrane transport	441	17	3.11×10^{-5}	2.38×10^{-3}
ossification	371	15	5.39×10^{-5}	3.73×10^{-3}
sensory perception of light stimulus	209	11	5.44×10^{-5}	3.73×10^{-3}
transporter complex	332	14	6.13×10^{-5}	4.00×10^{-3}
actin cytoskeleton	482	17	9.33×10^{-5}	5.80×10^{-3}
metal ion transmembrane transporter activity	440	16	1.06×10^{-4}	5.84×10^{-3}
connective tissue development	265	12	1.06×10^{-4}	5.84×10^{-3}
actinin binding	39	5	1.07×10^{-4}	5.84×10^{-3}
ion channel binding	123	8	1.36×10^{-4}	7.08×10^{-3}
extracellular matrix structural constituent	158	9	1.44×10^{-4}	7.19×10^{-3}
skeletal system morphogenesis	234	11	1.49×10^{-4}	7.19×10^{-3}
odontogenesis	126	8	1.60×10^{-4}	7.47×10^{-3}
cardiac chamber development	164	9	1.91×10^{-4}	8.57×10^{-3}
apical part of cell	375	14	2.20×10^{-4}	9.57×10^{-3}
pattern specification process	433	15	2.94×10^{-4}	1.23×10^{-2}
gland development	434	15	3.02×10^{-4}	1.23×10^{-2}
inorganic anion transport	177	9	3.36×10^{-4}	1.33×10^{-2}
cell-cell junction	441	15	3.57×10^{-4}	1.37×10^{-2}
SMAD binding	80	6	4.43×10^{-4}	1.57×10^{-2}
sensory system development	355	13	4.44×10^{-4}	1.57×10^{-2}
actomyosin structure organization	184	9	4.47×10^{-4}	1.57×10^{-2}
regulation of actin filament-based process	362	13	5.33×10^{-4}	1.83×10^{-2}
collagen trimer	87	6	6.93×10^{-4}	2.32×10^{-2}
actin-based cell projection	197	9	7.30×10^{-4}	2.38×10^{-2}
cell junction organization	285	11	7.93×10^{-4}	2.52×10^{-2}
monovalent inorganic cation transmembrane transporter activity	382	13	8.77×10^{-4}	2.72×10^{-2}
sensory perception of mechanical stimulus	163	8	9.04×10^{-4}	2.74×10^{-2}

somite development	92	6	9.30×10^{-4}	2.76×10^{-2}
muscle cell differentiation	338	12	9.60×10^{-4}	2.78×10^{-2}
ATPase activity	438	14	1.03×10^{-3}	2.85×10^{-2}
response to mechanical stimulus	207	9	1.04×10^{-3}	2.85×10^{-2}
detection of external stimulus	129	7	1.05×10^{-3}	2.85×10^{-2}
bone development	208	9	1.07×10^{-3}	2.85×10^{-2}
vacuolar lumen	170	8	1.19×10^{-3}	3.08×10^{-2}
detection of abiotic stimulus	132	7	1.21×10^{-3}	3.08×10^{-2}
positive regulation of ion transport	258	10	1.32×10^{-3}	3.27×10^{-2}
extracellular structure organization	400	13	1.33×10^{-3}	3.27×10^{-2}
cardiocyte differentiation	141	7	1.76×10^{-3}	4.12×10^{-2}
tongue development	20	3	1.77×10^{-3}	4.12×10^{-2}
ankyrin binding	20	3	1.77×10^{-3}	4.12×10^{-2}
cluster of actin-based cell projections	143	7	1.91×10^{-3}	4.35×10^{-2}
cellular component assembly involved in morphogenesis	106	6	1.93×10^{-3}	4.35×10^{-2}
neuromuscular process	107	6	2.03×10^{-3}	4.48×10^{-2}
embryonic organ development	423	13	2.18×10^{-3}	4.74×10^{-2}

Supplementary Table S8. Pathogenic rare variants on TTN and CLCN1.

Chr.	Position	Allele		Gene	Type	Pathogenicity			Alternative allele in 11 AIS patients										
		Ref.	Alt.			ClinVar	CADD	REVEL	1	2	3	4	5	6	7	8	9	10	11
2	178533203	C	T	TTN	nsSNV	Conflicting interpretations of pathogenicity	13.72	0.097	-	-	-	-	1	-	-	-	-	-	-
2	178560481	G	T	TTN	nsSNV	Conflicting interpretations of pathogenicity	18.23	0.669	-	-	-	-	-	-	-	-	-	-	1
2	178576090	C	T	TTN	nsSNV	Conflicting interpretations of pathogenicity	20.3	0.189	-	-	-	-	-	-	-	-	-	-	1
2	178605029	G	A	TTN	nsSNV	Conflicting interpretations of pathogenicity	23.2	0.401	-	1	-	-	-	-	-	-	-	-	-
2	178630312	C	A	TTN	nsSNV	Conflicting interpretations of pathogenicity	24.3	0.172	-	-	1	-	-	-	-	-	-	-	-
2	178709772	C	T	TTN	nsSNV	Conflicting interpretations of pathogenicity	24.6	0.24	-	-	-	1	-	-	-	-	-	-	-
2	178774941	G	C	TTN	nsSNV	Conflicting interpretations of pathogenicity	15.33	0.208	-	-	-	-	-	-	-	-	-	-	1
2	178802248	C	T	TTN	nsSNV	Conflicting interpretations of pathogenicity	24	0.499	-	-	-	-	-	-	1	-	-	-	-
2	178738312	C	T	TTN	nsSNV	Likely benign	22.6	0.814	-	-	-	-	-	1	-	-	-	-	-
7	143323375	G	A	CLCN1	nsSNV	Uncertain significance	32	0.843	-	-	-	-	-	-	1	-	-	-	-
7	143330810	G	A	CLCN1	nsSNV	Pathogenic/Likely pathogenic	29.5	0.9	-	-	-	-	-	-	-	-	-	-	1
7	143331616	G	A	CLCN1	nsSNV	-	35	0.915	-	-	-	-	-	-	-	-	-	-	1

1. Wang H, Wu Z, Zhuang Q, Fei Q, Zhang J, Liu Y, Wang Y, Ding Y, and Qiu G, *Association study of tryptophan hydroxylase 1 and arylalkylamine N-acetyltransferase polymorphisms with adolescent idiopathic scoliosis in Han Chinese.* Spine (Phila Pa 1976), 2008. **33**(20): p. 2199-203.
2. Khanshour AM, Kou I, Fan Y, Einarsdottir E, Makki N, et al., *Genome-wide meta-analysis and replication studies in multiple ethnicities identify novel adolescent idiopathic scoliosis susceptibility loci.* Hum Mol Genet, 2018. **27**(22): p. 3986-3998.
3. Kou I, Otomo N, Takeda K, Momozawa Y, Lu HF, et al., *Genome-wide association study identifies 14 previously unreported susceptibility loci for adolescent idiopathic scoliosis in Japanese.* Nat Commun, 2019. **10**(1): p. 3685.
4. Zhu Z, Tang NL, Xu L, Qin X, Mao S, et al., *Genome-wide association study identifies new susceptibility loci for adolescent idiopathic scoliosis in Chinese girls.* Nat Commun, 2015. **6**: p. 8355.
5. Li W, Li Y, Zhang L, Guo H, Tian D, et al., *AKAP2 identified as a novel gene mutated in a Chinese family with adolescent idiopathic scoliosis.* J Med Genet, 2016. **53**(7): p. 488-93.
6. Xu L, Xia C, Qin X, Sun W, Tang NL, Qiu Y, Cheng JC, and Zhu Z, *Genetic variant of BNC2 gene is functionally associated with adolescent idiopathic scoliosis in Chinese population.* Mol Genet Genomics, 2017. **292**(4): p. 789-794.
7. Zhao D, Qiu GX, Wang YP, Zhang JG, Shen JX, and Wu ZH, *Association between adolescent idiopathic scoliosis with double curve and polymorphisms of calmodulin1 gene/estrogen receptor-α gene.* Orthop Surg, 2009. **1**(3): p. 222-30.
8. Borysiak K, Janusz P, Andrusiewicz M, Chmielewska M, Kozinoga M, Kotwicki T, and Kotwicka M, *CHD7 gene polymorphisms in female patients with idiopathic scoliosis.* BMC Musculoskelet Disord, 2020. **21**(1): p. 18.
9. Sharma S, Gao X, Londono D, Devroy SE, Mauldin KN, et al., *Genome-wide association studies of adolescent idiopathic scoliosis suggest candidate susceptibility genes.* Hum Mol Genet, 2011. **20**(7): p. 1456-66.
10. Qiu XS, Tang NL, Yeung HY, Qiu Y, and Cheng JC, *Association study between adolescent idiopathic scoliosis and the DPP9 gene which is located in the candidate region identified by linkage analysis.* Postgrad Med J, 2008. **84**(995): p. 498-501.
11. Chen S, Zhao L, Roffey DM, Phan P, and Wai EK, *Association between the ESR1 -351A>G single nucleotide polymorphism (rs9340799) and adolescent idiopathic scoliosis: a systematic review and meta-analysis.* Eur Spine J, 2014. **23**(12): p. 2586-93.
12. Buchan JG, Alvarado DM, Haller GE, Cruchaga C, Harms MB, et al., *Rare variants in FBN1 and FBN2 are associated with severe adolescent idiopathic scoliosis.* Hum Mol Genet, 2014. **23**(19): p. 5271-82.
13. Sheng F, Xia C, Xu L, Qin X, Tang NL, Qiu Y, Cheng JC, and Zhu Z, *New Evidence Supporting the Role of FBN1 in the Development of Adolescent Idiopathic Scoliosis.* Spine (Phila Pa 1976), 2019. **44**(4): p. E225-e232.
14. Kou I, Takahashi Y, Johnson TA, Takahashi A, Guo L, et al., *Genetic variants in GPR126 are associated with adolescent idiopathic scoliosis.* Nat Genet, 2013. **45**(6): p. 676-9.
15. Guan M, Wang H, Fang H, Zhang C, Gao S, and Zou Y, *Association between IGF1 gene single nucleotide polymorphism (rs5742612) and adolescent idiopathic scoliosis: a meta-analysis.* Eur Spine J, 2017. **26**(6): p. 1624-1630.
16. Zhou S, Zhu Z, Qiu X, Wu W, Wang W, Liu Z, Lv F, and Qiu Y, *Association study of IL-17RC, CHL1, DSCAM and CNTNAP2 genes polymorphisms with adolescent*

idiopathic scoliosis susceptibility in a Chinese Han population. Stud Health Technol Inform, 2012. **176**: p. 47-51.
17. Zhou S, Qiu XS, Zhu ZZ, Wu WF, Liu Z, and Qiu Y, *A single-nucleotide polymorphism rs708567 in the IL-17RC gene is associated with a susceptibility to and the curve severity of adolescent idiopathic scoliosis in a Chinese Han population: a case-control study.* BMC Musculoskelet Disord, 2012. **13**: p. 181.
18. Gao J, Zhang L, Liu Z, Yao S, and Gao S, *[Correlation analysis between interleukin 6 polymorphism and adolescent idiopathic scoliosis susceptibility and bracing effectiveness].* Zhongguo Xiu Fu Chong Jian Wai Ke Za Zhi, 2018. **32**(6): p. 678-684.
19. Liu S, Wu N, Zuo Y, Zhou Y, Liu J, et al., *Genetic Polymorphism of LBX1 Is Associated With Adolescent Idiopathic Scoliosis in Northern Chinese Han Population.* Spine (Phila Pa 1976), 2017. **42**(15): p. 1125-1129.
20. Cao Y, Min J, Zhang Q, Li H, and Li H, *Associations of LBX1 gene and adolescent idiopathic scoliosis susceptibility: a meta-analysis based on 34,626 subjects.* BMC Musculoskelet Disord, 2016. **17**: p. 309.
21. Liu Z, Wang F, Xu LL, Sha SF, Zhang W, et al., *Polymorphism of rs2767485 in Leptin Receptor Gene is Associated With the Occurrence of Adolescent Idiopathic Scoliosis.* Spine (Phila Pa 1976), 2015. **40**(20): p. 1593-8.
22. Zhu Z, Xu L, Leung-Sang Tang N, Qin X, Feng Z, et al., *Genome-wide association study identifies novel susceptible loci and highlights Wnt/beta-catenin pathway in the development of adolescent idiopathic scoliosis.* Hum Mol Genet, 2017. **26**(8): p. 1577-1583.
23. Zhou T, Chen C, Xu C, Zhou H, Gao B, et al., *Mutant MAPK7-Induced Idiopathic Scoliosis is Linked to Impaired Osteogenesis.* Cell Physiol Biochem, 2018. **48**(3): p. 880-890.
24. Gao W, Chen C, Zhou T, Yang S, Gao B, et al., *Rare coding variants in MAPK7 predispose to adolescent idiopathic scoliosis.* Hum Mutat, 2017. **38**(11): p. 1500-1510.
25. Zhang H, Zhao S, Zhao Z, Tang L, Guo Q, Liu S, and Chen L, *The association of rs1149048 polymorphism in matrilin-1(MATN1) gene with adolescent idiopathic scoliosis susceptibility: a meta-analysis.* Mol Biol Rep, 2014. **41**(4): p. 2543-9.
26. Bae JW, Cho CH, Min WK, and Kim UK, *Associations between matrilin-1 gene polymorphisms and adolescent idiopathic scoliosis curve patterns in a Korean population.* Mol Biol Rep, 2012. **39**(5): p. 5561-7.
27. Zhao J, Yang M, and Li M, *Association of IL-6 and MMP-3 gene polymorphisms with susceptibility to adolescent idiopathic scoliosis: a meta-analysis.* J Genet, 2016. **95**(3): p. 573-9.
28. Yang P, Liu H, Lin J, and Yang H, *The Association of rs4753426 Polymorphism in the Melatonin Receptor 1B (MTNR1B) Gene and Susceptibility to Adolescent Idiopathic Scoliosis: A Systematic Review and Meta-analysis.* Pain Physician, 2015. **18**(5): p. 419-31.
29. Xu L, Xia C, Sun W, Qin X, Qiu Y, and Zhu Z, *Genetic Polymorphism of NUCKS1 Is Associated With the Susceptibility of Adolescent Idiopathic Scoliosis.* Spine (Phila Pa 1976), 2017. **42**(21): p. 1629-1634.
30. Xu L, Sheng F, Xia C, Qin X, Tang NL, Qiu Y, Cheng JC, and Zhu Z, *Genetic Variant of PAX1 Gene Is Functionally Associated With Adolescent Idiopathic Scoliosis in the Chinese Population.* Spine (Phila Pa 1976), 2018. **43**(7): p. 492-496.
31. Sharma S, Londono D, Eckalbar WL, Gao X, Zhang D, et al., *A PAX1 enhancer locus is associated with susceptibility to idiopathic scoliosis in females.* Nat Commun, 2015. **6**: p. 6452.

32. Qin X, He Z, Yin R, Qiu Y, and Zhu Z, *Abnormal paravertebral muscles development is associated with abnormal expression of PAX3 in adolescent idiopathic scoliosis.* Eur Spine J, 2020. **29**(4): p. 737-743.
33. Xu L, Sheng F, Xia C, Li Y, Feng Z, Qiu Y, and Zhu Z, *Common Variant of POC5 Is Associated With the Susceptibility of Adolescent Idiopathic Scoliosis.* Spine (Phila Pa 1976), 2018. **43**(12): p. E683-e688.
34. Patten SA, Margaritte-Jeannin P, Bernard JC, Alix E, Labalme A, et al., *Functional variants of POC5 identified in patients with idiopathic scoliosis.* J Clin Invest, 2015. **125**(3): p. 1124-8.
35. Wang WJ, Sun C, Liu Z, Sun X, Zhu F, Zhu ZZ, and Qiu Y, *Transcription factor Runx2 in the low bone mineral density of girls with adolescent idiopathic scoliosis.* Orthop Surg, 2014. **6**(1): p. 8-14.
36. Yang T, Xu JZ, Jia QZ, Guo H, Luo F, Ye Q, and Bai Y, *[Comparative analysis of sequence alignment of SH3GL1 gene as a disease candidate gene of adolescent idiopathic scoliosis].* Zhonghua Wai Ke Za Zhi, 2010. **48**(6): p. 435-8.
37. Yang T, Jia Q, Guo H, Xu J, Bai Y, Yang K, Luo F, Zhang Z, and Hou T, *Epidemiological survey of idiopathic scoliosis and sequence alignment analysis of multiple candidate genes.* Int Orthop, 2012. **36**(6): p. 1307-14.
38. Haller G, McCall K, Jenkitkasemwong S, Sadler B, Antunes L, et al., *A missense variant in SLC39A8 is associated with severe idiopathic scoliosis.* Nat Commun, 2018. **9**(1): p. 4171.
39. Qiao J, Xiao L, Xu L, Qian B, Zhu Z, and Qiu Y, *Genetic Variant of SOCS3 Gene is Functionally Associated With Lumbar Adolescent Idiopathic Scoliosis.* Clin Spine Surg, 2018. **31**(3): p. E193-e196.
40. Ryzhkov, II, Borzilov EE, Churnosov MI, Ataman AV, Dedkov AA, and Polonikov AV, *Transforming growth factor beta 1 is a novel susceptibility gene for adolescent idiopathic scoliosis.* Spine (Phila Pa 1976), 2013. **38**(12): p. E699-704.
41. Andrusiewicz M, Harasymczuk P, Janusz P, Biecek P, Żbikowska A, Kotwicka M, and Kotwicki T, *TIMP2 Polymorphisms Association With Curve Initiation and Progression of Thoracic Idiopathic Scoliosis in the Caucasian Females.* J Orthop Res, 2019. **37**(10): p. 2217-2225.
42. Jiang J, Qian B, Mao S, Zhao Q, Qiu X, Liu Z, and Qiu Y, *A promoter polymorphism of tissue inhibitor of metalloproteinase-2 gene is associated with severity of thoracic adolescent idiopathic scoliosis.* Spine (Phila Pa 1976), 2012. **37**(1): p. 41-7.
43. Wang Y, Cui ZQ, Luo TB, and Liu L, *Correlations of VDR and VDBP genetic polymorphisms with susceptibility to adolescent idiopathic scoliosis and efficacy of brace treatment.* Genomics, 2016. **108**(5-6): p. 194-200.
44. Yin X, Wang H, Guo J, Zhang L, Zhang Y, Li L, and Hou S, *Association of vitamin D receptor BsmI rs1544410 and ApaI rs7975232 polymorphisms with susceptibility to adolescent idiopathic scoliosis: A systematic review and meta-analysis.* Medicine (Baltimore), 2018. **97**(2): p. e9627.
45. Suh KT, Eun IS, and Lee JS, *Polymorphism in vitamin D receptor is associated with bone mineral density in patients with adolescent idiopathic scoliosis.* Eur Spine J, 2010. **19**(9): p. 1545-50.
